# Twisted intramolecular charge transfer of nitroaromatic push–pull chromophores

**DOI:** 10.1038/s41598-022-10565-6

**Published:** 2022-04-21

**Authors:** Sebok Lee, Myungsam Jen, Taehyung Jang, Gisang Lee, Yoonsoo Pang

**Affiliations:** Department of Chemistry, Gwangju Institute of Science and Technology, 123 Cheomdangwagi-ro, Buk-gu, Gwangju, 61005 Republic of Korea

**Keywords:** Physical chemistry, Excited states

## Abstract

The structural changes during the intramolecular charge transfer (ICT) of nitroaromatic chromophores, 4-dimethylamino-4′-nitrobiphenyl (DNBP) and 4-dimethylamino-4′-nitrostilbene (DNS) were investigated by femtosecond stimulated Raman spectroscopy (FSRS) with both high spectral and temporal resolutions. The kinetically resolved Raman spectra of DNBP and DNS in the locally-excited and charge-transferred states of the S_1_ state appear distinct, especially in the skeletal vibrational modes of biphenyl and stilbene including ν_8a_ and ν_C=C_. The ν_8a_ of two phenyls and the ν_C=C_ of the central ethylene group (only for stilbene), which are strongly coupled in the planar geometries, are broken with the twist of nitrophenyl group with the ICT. Time-resolved vibrational spectroscopy measurements and the time-dependent density functional theory simulations support the ultrafast ICT dynamics of 220–480 fs with the twist of nitrophenyl group occurring in the S_1_ state of the nitroaromatic chromophores. While the ICT of DNBP occurs via a barrier-less pathway, the ICT coordinates of DNS are strongly coupled to several low-frequency out-of-phase deformation modes relevant to the twist of the nitrophenyl group.

## Introduction

Intramolecular charge transfer (ICT) is one of the fundamental photophysical processes in many chemical and biological systems and has been extensively investigated experimentally and theoretically^1–5^. The dyes with the strong ICT characters often show the large Stokes shifts and abrupt quantum yield changes with the ICT, and lead to the charge separation and charge transfer in many applications, including oxidation and reduction reactions in natural photosynthesis, photocatalytic metal complexes, charge injection in dye-sensitized solar cells, etc^1,6–15^. Upon photoexcitation to the excited states, redistribution of electrons around all the nuclei of a molecule occurs. When a charge transfer occurs between the electron donor and electron acceptor groups often connected by a conjugated π bridge, the chromophore exhibits a large increase in the dipole moment in the excited states.

The ICT dynamics of *push–pull* chromophores and molecular complexes have been extensively studied by numerous time-resolved spectroscopy and theoretical methods^4,16–26^. Most of these works focused on the photophysical properties, including absorption and emission, and the structural changes of the chromophores or complexes in the excited state such as the twist of electron donor or acceptor groups have been often suggested based on the changes in the electronic structures and theoretical simulations results^4,5,18,27–31^. However, the structural changes of chromophores during the ICT have rarely been directly evidenced by the time-resolved structural probes including infrared absorption and Raman scattering^20,32–35^. For example, 4-dicyanomethylene-2-methyl-6*p*-dimethyl aminostyryl-4*H*-pyran (DCM) which shows a strong ICT character in the excited state has sought wide applications including laser dyes, OLED emitters, optical chemosensors, etc. due to the high quantum yield and photostability^36–38^. Twist of the electron-donating dimethylamino group during the ICT has been proposed by several theoretical and experimental investigations, but disputed with a proposal of “planar” ICT structure^32,39–42^. Recently, we investigated the structural changes of DCM before and after the ICT process by femtosecond stimulated Raman spectroscopy (FSRS), where the solvent polarity dependent ICT dynamics of DCM has been evidenced in the major vibrational modes including the ν_8a_ mode^32^. From the multi-mode spectral difference between the locally-excited (LE) and ICT states of DCM, the twist of the dimethylamino group was confirmed. The ICT dynamics of 1.0 ps time constant was retrieved from FSRS measurements compatible with previous observations with transient absorption and fluorescence spectroscopy^32,41,43,44^.

**Figure 1:**
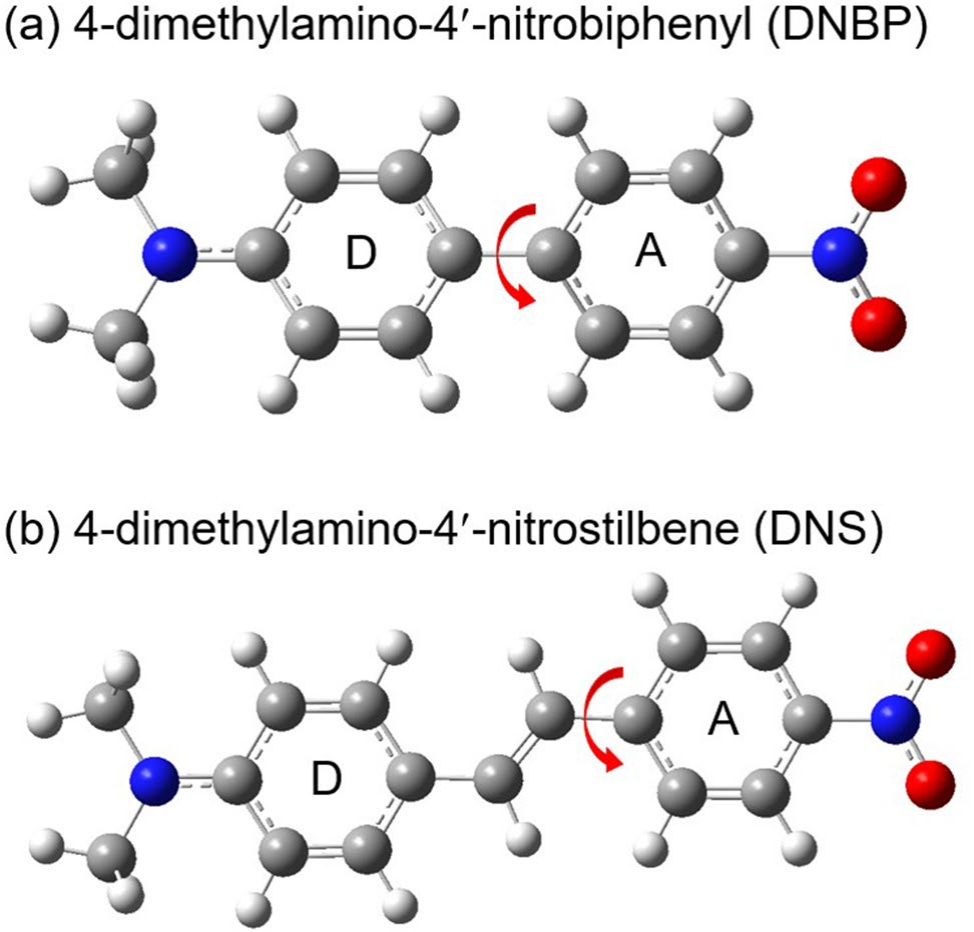
Molecular structures of nitroaromatic *push–pull* chromophores, (**a**) 4-dimethylamino-4′-nitrobiphenyl (DNBP) and (**b**) 4-dimethylamino-4′-nitrostilbene (DNS) optimized in the ground state by the DFT simulations at the B3LYP/6-311G(d,p) level with the polarized continuum model (PCM) for CHCl_3_.

The ICT dynamics of the *push–pull* chromophores with a nitro group, 4-dimethylamino-4′-nitrobiphenyl (DNBP) and 4-dimethylamino-4′-nitrostilbene (DNS) have also been investigated in several experimental and theoretical studies^40,45–51^. As shown in Fig. 1, these nitroaromatic chromophores share the electron-donating dimethylamino and the electron-withdrawing nitro group at both ends of each molecule with the π-conjugated backbone of biphenyl for DNBP and stilbene for DNS. The electron-withdrawing nitro group induces the strong ICT character in the excited states and large Stokes shifts in many *push–pull* chromophores^18,21,24,45,47,48,52,53^. Twist of the nitro group has been commonly suggested for the ICT of small nitroaromatic molecules by time-resolved absorption and emission measurements and theoretical simulations^18,21,24,52–54^. The twisted structure with the rotation of nitrophenyl group has been proposed by time-resolved absorption and emission measurements and semi-empirical and time-dependent density functional theory (TDDFT) simulations^40,48,50^. However, the rotation of the nitro group has also been suggested for the ICT of DNS from the excited state dynamics comparison between DNS and a constrained model compound for nitrophenyl rotation^47,49^. Similarly, twisted ICT structure of DNBP with the nitrophenyl rotation has been proposed by time-resolved electronic spectroscopy and theoretical simulations^45,46^. Nonetheless, the molecular structures of DNS and DNBP in the ICT states are not yet confirmed by any experimental evidence. The ultrafast electronic transitions with strong Stokes shifts in emission are generally considered evidence of structural changes such as the internal rotation of a certain moiety, along with theoretical verifications by TDDFT or semi-empirical calculations. The excited state infrared and Raman spectra of DNS have also been reported by time-resolved vibrational spectroscopy^55,56^. However, the excited state spectra of DNS in the S_1_/ICT state were not resolved clearly due to low temporal and spectral resolutions of picosecond time-resolved infrared absorption and picosecond coherent anti-Stokes Raman spectroscopy.

In this work, FSRS with both high spectral (< 10 cm^−1^) and temporal (< 50 fs) resolutions was used to investigate the ultrafast structural dynamics of nitroaromatic *push–pull* chromophores during the ICT process in the excited state^57–59^. The FSRS has been successfully applied in the observation of various ultrafast dynamics in the excited states, including the intra- and inter-molecular proton transfers^60–63^, charge transfers^33,64,65^, etc^66,67^. The population dynamics and the peak shifts of the major vibrational modes in the finger-print frequency range would provide sufficient structural information of chromophores in the excited states. The structural changes during a specific excited state process, including proton transfers and charge transfers, or the vibrational relaxation in the excited state potential surfaces can better be estimated from the chromophore’s instantaneous Raman spectrum.

## Results and discussion

Steady-state absorption and emission spectra of DNBP and DNS in the solvents of various polarities are shown in Fig. S1 in the Supplementary Information. While the absorption spectra of DNBP and DNS show the solvatochromic shifts (373–409 nm for DNBP and 413–453 nm for DNS) depending on the solvent polarity, the emission bands show large Stokes shifts of 8010–11,800 cm^−1^ for DNBP and 7390–11,300 cm^−1^ for DNS in polar solvents. The strong ICT characters of DNBP and DNS in the excited states have also been reported^45,46,48,49^.

**Figure 2:**
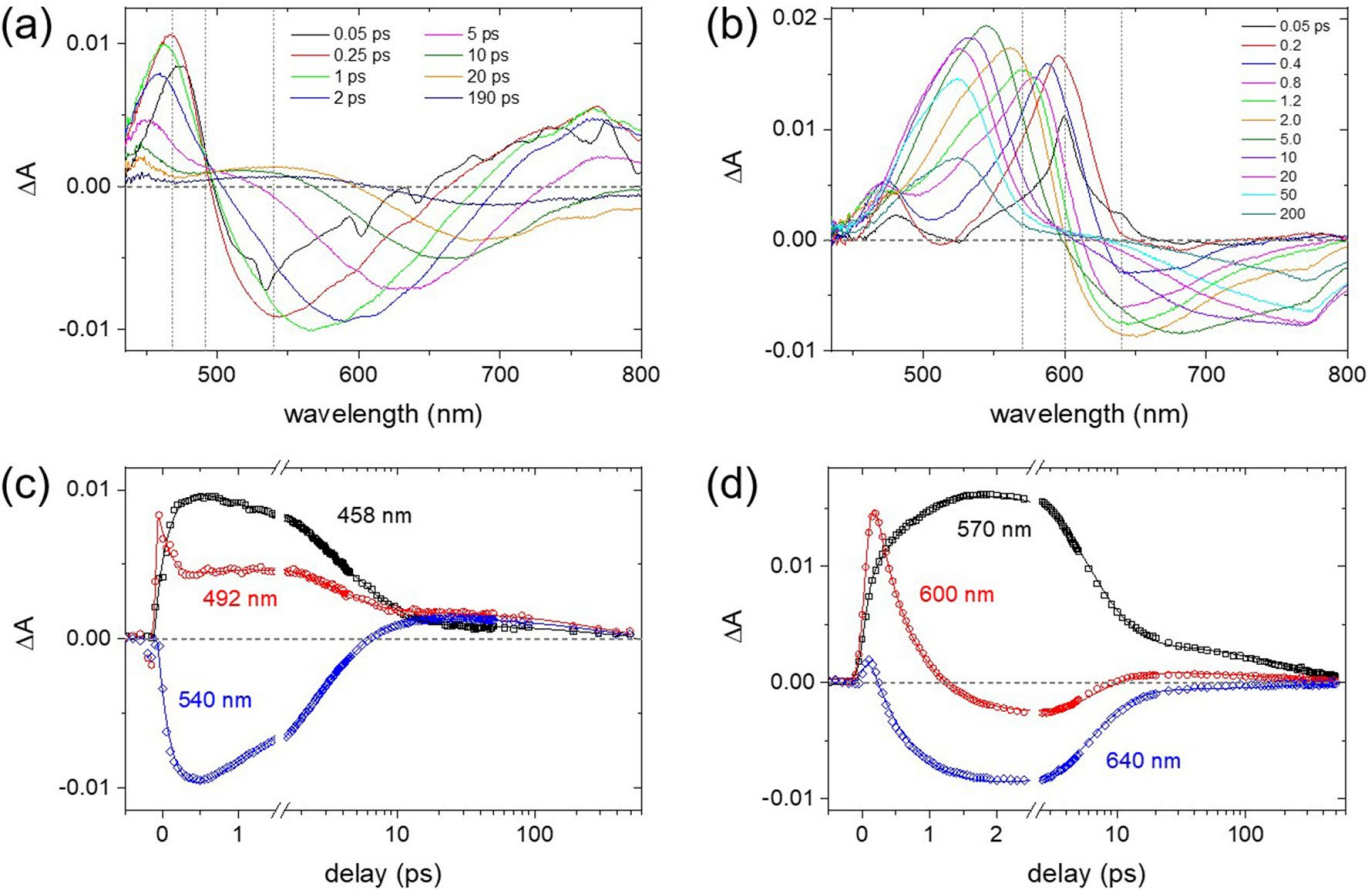
Transient absorption spectra of (**a**) DNBP and (**b**) DNS in CHCl_3_ solution obtained with the 403 nm excitation, the excited state kinetics for the locally-excited (LE) and charge-transferred (CT) states of (**c**) DNBP and (**d**) DNS.

Transient absorption results of DNBP and DNS in CHCl_3_ with 403 nm excitation were shown in Fig. 2, where the excited state kinetics were fit to the Gaussian-convoluted exponential functions^32,62^. The ICT of DNBP in polar solvents appears to be a barrierless transition with a strong dependence on the solvent polarity or viscosity^45,46,68,69^. The ultrafast ICT dynamics of DNBP with the time constant of 220 fs is represented by the excited state absorption (ESA) bands at 492 nm for the LE state and 458 nm for the charge-transferred (CT) state shown in Fig. 2c. The ESA and stimulated emission (SE) bands of DNBP probed at 458 and 540 nm, respectively, also show the solvational dynamics (3.5 ps) and the population decay (170 ps) of the CT state. The solvation dynamics of DNBP in the CT state originating from the dipolar interactions with solvents exhibit the strong red-shift (540 → 695 nm) in the SE bands and the blue-shift (467 → 447 nm) in the ESA band, as shown in Fig. 2a. The ultrafast ICT dynamics of 0.4–0.7 ps have been observed for DNBP in the polar solvents of acetonitrile and methanol, where the twist of biphenyl group has been suggested for the ICT in the S_1_ excited state^45,46^.

The excited-state dynamics of DNS also appear strongly dependent on the solvent polarity^40,47–50^. The ESA bands of DNS also show the ultrafast internal conversion of 440 fs between the LE band at 600 nm and the CT state at 570 nm, as shown in Fig. 2d. The ESA and SE bands of DNS in the ICT state show strong spectral shifts up to 20 ps, which is interpreted as the dipolar solvation dynamics in the ICT state. The excited-state kinetics of the ICT bands in Fig. 2d show the biexponential dynamics of 1.1 and 4.8 ps in addition to the population decay of 230 ps. Although the transient absorption provides extensive information on the electronic transitions of the excited state created by the photoexcitation, the detailed information on the structural changes of chromophores in the excited state upon the ICT and the subsequent solvation dynamics would not be available.

**Figure 3:**
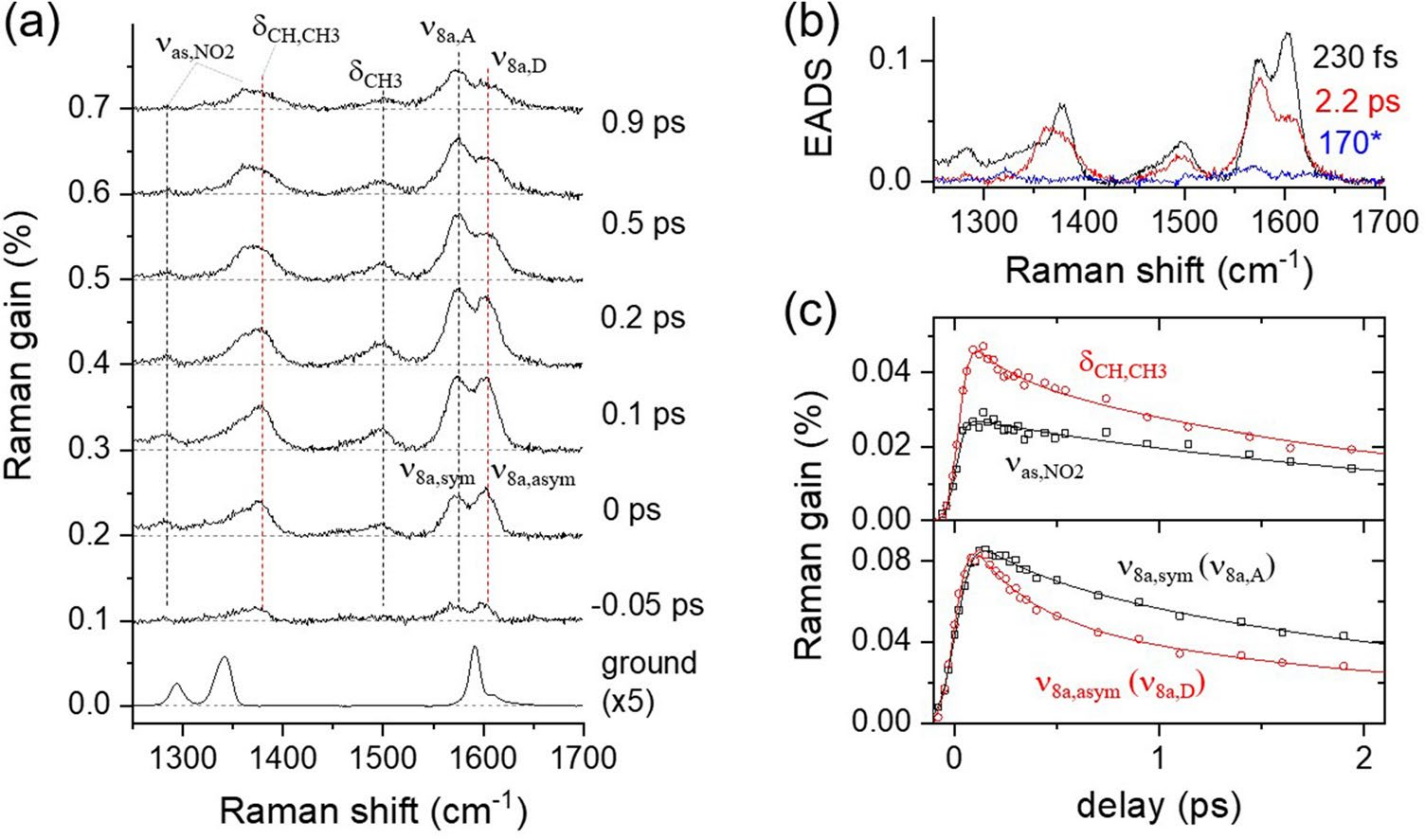
Femtosecond stimulated Raman spectroscopy (FSRS) of 4-dimethylamino-4′-nitrobiphenyl (DNBP) with 403 nm excitation; (**a**) time-resolved Raman spectra compared to the ground spectrum, (**b**) evolution-associated difference spectra (EADS) from the global analysis of excited state Raman spectra, (**c**) kinetic traces for the major vibrational modes of ν_as,NO2_ (1361 cm^−1^) and δ_CH,CH3_ (1378 cm^−1^), ν_8a,sym_ (1574 cm^−1^), and ν_8a,asym_ (1602 cm^−1^) modes.

The FSRS results of DNBP in CHCl_3_ with the 403 nm excitation are shown in Fig. 3a. The excited-state Raman spectra of DNBP are distinct from the ground state spectrum compared together. Vibrational assignments for the ground and excited-state Raman spectra of DNBP were made based on the DFT and TDDFT simulations, and the twist of the nitrophenyl group is expected in the ICT in the excited state^45^. The details of the optimized structures and vibrational spectra of DNBP in the ground and excited state are available in the Supplementary Information. In the ground state, two phenyl rings of DNBP appear more or less parallel to each other, as shown in Fig. 1a; the dihedral angle between the biphenyl is 30.4°. Thus the ν_8a_ modes of two phenyls are strongly coupled into the symmetric and antisymmetric modes of ν_8a_ appearing at 1592 and 1610 cm^−1^ (shoulder), respectively, in the ground state spectrum. Other major vibrational bands of DNBP in the ground state spectrum at 1294 and 1342 cm^−1^ are assigned as the ν_C-C_ + δ_CH_ and ν_s,NO2_ + δ_CH_ modes, respectively^70,71^.

In the excited-state Raman spectra of DNBP in Fig. 3a, ν_8a_ modes of biphenyls appeared red-shifted to 1574 and 1602 cm^−1^, and the relative intensity of the asymmetric mode at an initial time delay of 0 ps largely increases from that of the ground state. The spectral shifts and changes in the relative intensity of the ν_8a_ modes show that the electronic densities around the nuclei are changed abruptly upon the photoexcitation to the excited state^72–75^. Other major vibrational modes of DNBP appearing in the excited state spectra are the asymmetric NO_2_ stretching ν_as,NO2_ appearing at 1283 and 1361 cm^−1^, respectively, and the δ_CH,CH3_ and δ_CH3_ modes at 1378 and 1499 cm^−1^, respectively. The intensity ratio between the ν_8a,sym_ and ν_8a,asym_ modes shows an ultrafast increase in the excited state, and the intensity of ν_8a,sym_ at 1574 cm^−1^ becomes larger than that of ν_8a,asym_ at 1602 cm^−1^ at 0.2 ps or later time delays. In other words, the ν_8a,asym_ mode shows a fast decay of 0.28 ps which is absent in the dynamics of the ν_8a,sym_ mode as compared in Fig. 3c. The intensity ratio between these vibrational modes shown in Fig. S16a in the Supplementary Information clearly indicates the appearance and absence of the ultrafast dynamics in these skeletal vibrational modes of DNBP.

**Figure 4:**
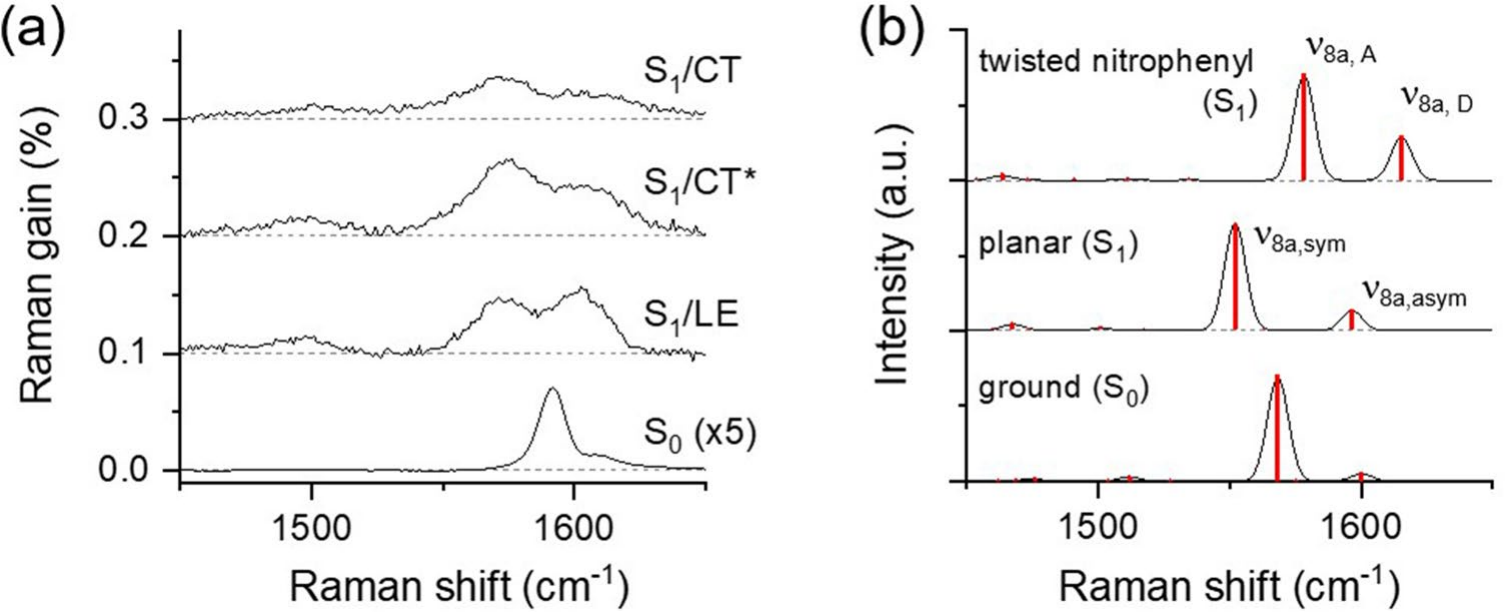
(**a**) Femtosecond stimulated Raman spectra of DNBP in CHCl_3_ solution with 403 nm excitation. Each spectrum representing the S_1_/LE, S_1_/CT*, and S_1_/CT state was obtained at the time delays of 0.0, 0.9, and 2.9 ps. (**b**) Simulated Raman spectra of DNBP in the ground state, and the S_1_ excited states with the planar and twisted nitrophenyl geometry. Approximate bandwidths of 10 cm^−1^ were used for the simulated Raman spectra.

The spectral changes in the ν_8a_ modes of DNBP with the twist of the nitrophenyl during the ICT in the S_1_ excited state can be understood from the results of the TDDFT simulations^45^. Figure 4 compares the excited state Raman spectra of DNBP in CHCl_3_ and the simulated Raman spectra in the LE and ICT states with the planar and twisted nitrophenyl geometries, respectively. The S_1_ minimum was found in the TDDFT simulations at the B3LYP/6-311G(d,p) level with the further twist of the dihedral angle between the biphenyl to 89.5° and two coupled vibrational modes of ν_8a,sym_ and ν_8a,asym_ with the planar geometry are separated into ν_8a,D_ and ν_8a,A_. The vibrational frequencies of the ν_8a_ modes of biphenyl are expected to blue-shift by 20–25 cm^−1^, and the intensity ratio between the ν_8a,A_ and ν_8a,D_ modes appears slightly different from that between the ν_8a,sym_ and ν_8a,asym_ modes based on the TDDFT simulations for the twist of nitrophenyl group. However, no major frequency changes were observed in the ν_8a_ modes of DNBP from FSRS measurements except the small blue-shift of 5 cm^−1^ in the ν_8a,asym_ (ν_8a,D_) mode. Nonetheless, the abrupt intensity changes between ν_8a,sym_ and ν_8a,asym_ shown in Fig. 4a can only be explained by the major structural changes of the biphenyl backbone, which occurs on the ultrafast time scale of 0.28 ps. It is generally accepted that the frequencies and Raman optical activities for the vibrational modes in the excited states are strongly dependent on the level of the DFT theory^76^. Further explanations of the experimental Raman spectra of DNBP in the twisted ICT may require more precise theoretical investigations, for example, adopting the basis sets with diffuse functions^77^.

Time-resolved Raman spectra of DNBP in the frequency range of 1250–1700 cm^−1^ were analyzed in a global analysis with a sequential decay model^78^, which also strongly supports the decoupling of two ν_8a_ modes of biphenyl upon the twist of biphenyl group in the S_1_ excited state. Three kinetic components were retrieved from the time-resolved Raman data of DNBP. The evolution-associated difference spectra (EADS) with the time constant of 230 fs shown in Fig. 3b represents the LE spectrum, while the 2.2 and 170 ps represent the spectra of DNBP in the vibrationally hot CT* and relaxed CT states, respectively.

Figure 3c shows the kinetic traces for the major vibrational modes of DNBP in the excited state. The ultrafast ICT dynamics of 230 fs were commonly observed in the ν_8a,asym_ (1602 cm^−1^) and δ_CH,CH3_ (1378 cm^−1^) modes while the ν_8a,sym_ (1574 cm^−1^) and ν_as,NO2_ (1283 cm^−1^) modes only show the vibrational relaxation or solvation dynamics of 2.2 ps in the ICT state. Since the vibrational modes of ν_8a,sym_ and ν_as,NO2_ showed no major frequency shifts in FSRS measurements, the 2.2 ps dynamics observed from these modes are considered to represent the dipolar solvation dynamics of CHCl_3_. Based on the time-resolved electronic and vibrational spectroscopic results, the excited-state dynamics of DNBP in CHCl_3_ can be summarized as the ICT process of 220 fs with the twist of nitrophenyl, the solvent dynamics in the CT state of 2.2–3.5 ps, and the population decay of 170 ps.

**Figure 5:**
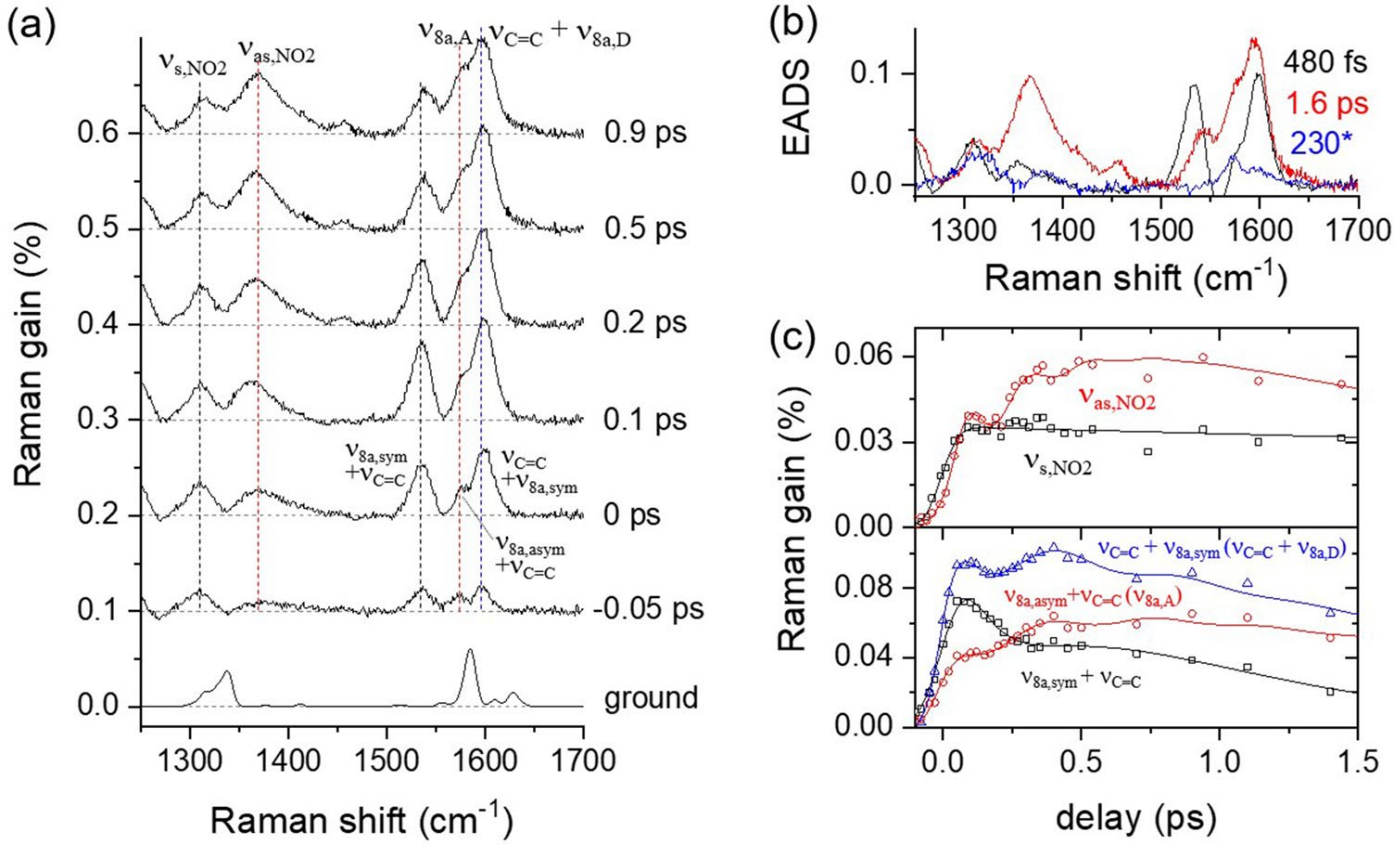
Femtosecond stimulated Raman spectroscopy (FSRS) of 4-dimethylamino-4′-nitrostilbene (DNS) with 403 nm excitation; (**a**) time-resolved Raman spectra compared to the ground state spectrum, (**b**) evolution-associated difference spectra (EADS) from the global analysis of excited state Raman spectra, (**c**) kinetic traces for the major vibrational modes of ν_NO2_, ν_C=C_, and ν_8a_ modes of phenyl. The vibrational modes outside the parentheses denote the DNS modes with the planar geometry and those inside the parentheses represent the vibrational modes with the twisted nitrophenyl geometry.

The excited-state Raman spectra of DNS in CHCl_3_ with the 403 nm excitation are shown in Fig. 5a. The excited-state Raman spectra are distinct from the ground state spectrum displayed together. Vibrational assignments for the ground and excited-state Raman bands of DNS were based on the DFT and TDDFT simulations and the previous reports with picosecond resonance Raman spectroscopy^55,56,79^. The details of the DFT and TDDFT simulations of DNS including the optimized structures and Raman spectra are available in the Supplementary Information. The overall planar geometry of DNS shown in Fig. 1b was obtained from the DFT simulations in the ground state. The major vibrational modes of DNS in the ground state includes the ν_8b, D_ at 1556 cm^−1^, ν_8a,sym_ + ν_C=C_ at 1585 cm^−1^, ν_8a,asym_ + ν_C=C_ at 1610 cm^−1^, and ν_C=C_ + ν_8a,sym_ at 1629 cm^−1^. It is interesting to note that the skeletal vibrational modes ν_8a_ of two phenyl groups and ethylenic ν_C=C_ between two phenyls are strongly coupled to each other, resulting in the four-band spectral pattern in the frequency range of 1550–1650 cm^−1^. Other major vibrational modes of DNS in the ground state include δ_CH_ and ν_s,NO2_ + δ_CH_ modes appearing at 1316 and 1338 cm^−1^, respectively, as shown in Fig. 5a. Upon photoexcitation, ν_8a_ and ν_C=C_ modes of DNS in the frequency range of 1560–1650 cm^−1^ appear red-shifted to 1535 cm^-1^ (ν_8a,sym_ + ν_C=C_), 1575 cm^−1^ (ν_8a,asym_ + ν_C=C_), and 1598 cm^−1^ (ν_C=C_ + ν_8a,sym_) with respect to the ground-state frequencies in the early time delays.

The relative intensity of the ν_8a,sym_ + ν_C=C_ mode shows an ultrafast decrease in 0.5–1.0 ps while the ν_8a,asym_ + ν_C=C_ (ν_8a,A_) mode shows an increase in intensity, as compared in Fig. 5c. The intensity changes in these vibrational modes of DNS appear more clear in the plot of the intensity between two modes shown in Fig. S16b in the Supplementary Information. It is also interesting to note that the ν_C=C_ + ν_8a,sym_ mode maintains its initial intensity in 1 ps. Three kinetic components retrieved from the time-resolved Raman data of DNS by the global analysis with a sequential decay model are shown in Fig. 5b^78^. The EADS with the 480 fs component represents the LE spectrum of DNS, while the 1.6 and 230 ps components represent the vibrationally hot and relaxed CT spectra, respectively. The ultrafast intensity changes (480 fs) in the ν_8a,sym_ + ν_C=C_ and ν_8a,asym_ + ν_C=C_ modes of DNS may provide the key information to the structural changes of DNS upon the ICT in the S_1_ excited state, which is compatible to the ICT dynamics (440 fs) observed in the transient absorption measurements. Besides, the ν_s,NO2_ at 1310 cm^−1^ and ν_as,NO2_ at 1370 cm^−1^ appear in the excited state Raman spectra of DNS. While the ν_s,NO2_ mode shows no significant intensity changes during the ICT, the ν_as,NO2_ mode shows a signal increase of 480 fs for the ICT dynamics in the S_1_ excited state.

The TDDFT simulations estimate the structural changes of DNS in the S_1_/ICT state from the ground state geometry. The previous theoretical works on DNS in the excited states have shown that the excited state dynamics of DNS are strongly dependent on solvent polarity, and several excited-state geometries, including the twisted dimethylamino, nitro, and nitrophenyl groups, were proposed for the S_1_/ICT state^40,48,49,80,81^. The overall planar geometry of DNS is expected in the S_1_ minimum from the pseudo-potential simulations shown in Fig. S7b in the Supplementary Information, while the relative energies of DNS with the twisted nitro and nitrophenyl groups are higher than that with the planar geometry by 0.47 and 0.15 eV, respectively. However, the excited state simulations results by the TDDFT methods are often inaccurate and strongly dependent on the level of theory, especially for molecules with strong charge transfer characters^80,82^. Therefore, the search for the local minima in the S_1_ excited state was performed by the further optimizations in the S_1_ excited state with possible structural changes, including the twisting of dimethylamino, nitro, or nitrophenyl group. Then, the structural changes accompanying the ICT in the S_1_ excited state can be justified by comparing the experimental Raman spectra obtained from FSRS measurements with the simulated Raman spectra obtained within the resultant local minima with the specific twisted molecular geometry of DNS in the S_1_ excited state. Table S2 in the Supplementary Information summarizes the structure optimizations of DNS in the S_1_ excited state.

**Figure 6:**
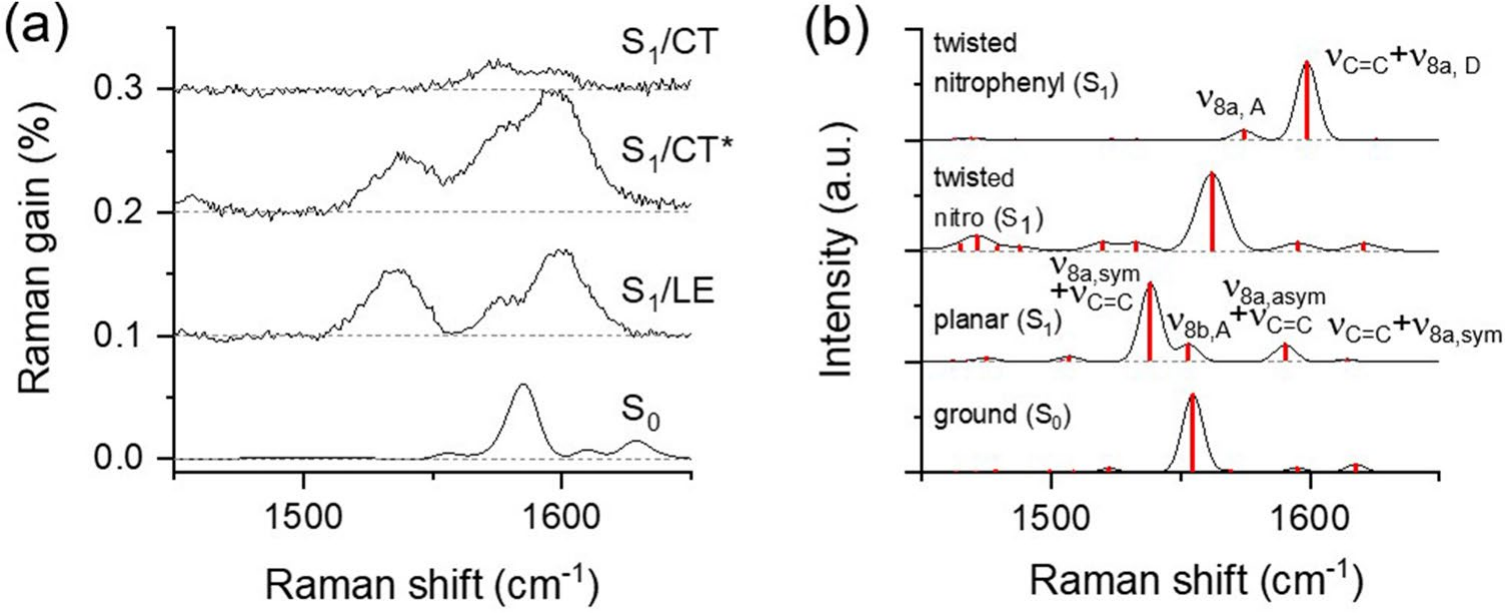
(**a**) Femtosecond stimulated Raman spectra of DNS in CHCl_3_ solution with 403 nm excitation. Each spectrum representing the S_1_/LE, S_1_/CT*, and S_1_/CT state was obtained at the time delays of 0.0, 0.9, and 9.9 ps. (**b**) simulated Raman spectra of DNS in the ground state, and the S_1_ excited states with the planar, twisted nitro, and twisted nitrophenyl geometry. Approximate bandwidths of 10 cm^−1^ were used for the simulated Raman spectra.

The local minima of DNS with the twisted nitro and nitrophenyl groups in the S_1_ excited state were found by the TDDFT simulations, where the relative energies of DNS with the twisted nitro and nitrophenyl groups appear slightly lower (0.03–0.06 eV) than the planar geometry. Figure 6 compares the ground and excited-state Raman spectra of DNS in CHCl_3_ with the simulated Raman spectra in the ground state, S_1_/LE, and S_1_/CT states. The simulated Raman spectrum of DNS with the planar geometry in the S_1_ excited state shows red-shifts of 5–15 cm^−1^ from the ground state frequencies in the major vibrational modes of ν_8a_ and ν_C=C_. The simulated spectrum of DNS with the twisted nitro geometry appears quite similar to the ground state spectrum in terms of the major skeletal vibrations of ν_8a_ and ν_C=C_ and blue-shifted from the spectrum with the planar geometry. On the other hand, the twist of the nitrophenyl group (the dihedral angle between the nitrophenyl and ethylene is 89.5°) induces substantial changes of the ν_8a_ and ν_C=C_ modes. As shown in Fig. 6b, the vibrational couplings between the ν_8a_ of phenyls and the ν_C=C_ of the central ethylene with the planar geometry are broken into the ν_8a,A_ and the ν_C=C_ + ν_8a,D_ with the twist of nitrophenyl. The TDDFT optimization of DNS with the twisted dimethylaminophenyl does not converge in a reasonable time window, and was not considered further.

The twist of nitrophenyl group appears to induce huge spectra changes in the skeletal vibrational modes of ν_8a_ and ν_C=C_ as shown in Fig. 6b. The disappearance of ν_8a,sym_ + ν_C=C_ at 1535 cm^−1^, and the relative increases in the vibrational modes of ν_8a,A_ at 1578 and ν_C=C_ + ν_8a,D_ at 1598 cm^−1^ in the S_1_/CT spectra shown in Fig. 6a are all consistent to the theoretical estimation of the twist of nitrophenyl group by the TDDFT simulations. Further theoretical explorations, for example, by using more appropriate basis set with the diffuse functions are required for the evaluation of the experimental Raman results of DNS with the ICT dynamics in the S_1_ state. However, the substantial changes in the skeletal vibrational modes of stilbene including the disappearance of the major coupled vibrational modes of ν_8a,sym_ + ν_C=C_ and changes in the relative intensities of other skeletal vibrational modes of ν_8a_ and ν_C=C_ can only be explained with the major structural changes in the stilbene backbone. Based on the current TDDFT simulation results, the twist of nitrophenyl group is considered most probable for the structural changes accompanying the ICT dynamics in the S_1_ state.

The time-dependent excited-state Raman spectra of DNS in Figs. 5a and 6a are well understood with the ICT dynamics of 480 fs, which induces the structural changes of DNS, including the twist of the nitrophenyl group. In the S_1_/LE state with the planar geometry, the Raman spectrum of DNS shows the major skeletal vibrational modes of stilbene backbone including the ν_8a,sym_ + ν_C=C_ at 1535 cm^−1^ , ν_8a,asym_ + ν_C=C_ at 1575 cm^−1^, and ν_C=C_ + ν_8a,sym_ at 1598 cm^−1^. Ultrafast ICT dynamics of DNS with the twist of nitrophenyl group breaks up the vibrational coupling between the acceptor side phenyl and the rest of the molecule (dimethylaminophenyl and central ethylene group). The vibrational modes of DNS in the relaxed CT state are interpreted as the ν_8a,A_ at 1578 cm^−1^ and ν_C=C_ + ν_8a,D_ at 1598 cm^−1^.

Figure 5c compares the kinetics of the major vibrational modes of DNS in the excited state, where the strong coherent oscillations were fit by the sum of the Gaussian-convoluted exponential functions and the damped oscillation functions (see eq. S2 in the Supplementary Information). From the coherent oscillation signals in the major vibrational modes of DNS, the frequencies of 72, 83, and 146 cm^−1^ were retrieved by fast Fourier transformation (see Fig. S13 in the Supplementary Information). As shown in Fig. S14 in the Supplementary Information, the ν_C=C_ + ν_8a,sym_ and ν_as,NO2_ modes also show strong coherent oscillations in the center frequencies, where the similar frequencies of 79 and 146 cm^−1^, respectively, were obtained as those from the population dynamics of both vibrational modes. Coherent oscillations in the excited-state vibrational results often represent that the reaction coordinates of the ICT of DNS are strongly coupled to the low-frequency vibrational modes, for example, out-of-plane deformation vibrations relevant to the structural changes of the chromophore in the specific excited-state processes^59,63,65,83–87^. Figure S15 in the Supplementary Information lists the out-of-plane deformation modes of DNS in the S_1_ excited state with a planar geometry, which can be related to the twist of the nitrophenyl group upon the ICT in the excited state. Mathies and co-workers reported similar coherent oscillations in the population and the frequencies of several vibrational modes, including the ν_C-O_ and ν_C=N_ in the excited-state proton transfer of green fluorescent protein, which was interpreted as the strong coupling to the out-of-plane wagging vibration (120 cm^−1^) of the phenol ring^87^. Recently, we also reported strong coherent oscillations in the ν_ring_ and ν_C=O_ modes with the twisted ICT of 1-aminoanthraquinone, where the internal rotation of amino group is considered strongly coupled to the low-frequency out-of-deformation modes (96–194 cm^−1^) of the amino and adjacent carbonyl groups^65^. Anharmonic couplings between the high-frequency vibrational modes and the low-frequency bending modes along the reaction coordinates of the intermolecular charge transfer and ring-opening in the excited state have been thoroughly explored by two-dimensional FSRS works^88,89^.

Time-resolved Raman spectra of DNS have been previously reported by picosecond resonance Raman and coherent anti-Stokes Raman spectroscopy, where the appearances of 1626–1635 cm^−1^ and red-shifts of the ν_s,NO2_ to 1298–1299 cm^−1^ were interpreted as the twist of the nitro group^55,79^. The ν_s,NO2_ in the S_1_ excited state was not observed in our FSRS measurements. However, the appearance of the strong skeletal vibrational mode at 1626–1635 cm^−1^ would be better related to the structural changes of the stilbene backbone. The detailed vibrational features of DNS, including the vibrational couplings between the ν_8a_ of phenyls and the ν_C=C_ of ethylene, can provide crucial information on the structural changes of the stilbene backbone in the excited states. Recently, the ICT dynamics of a similar *push–pull* chromophore to DNS, 4-(dicyanomethylene)-2-methyl-6-(4-dimethylaminostyryl)-4*H*-pyran (DCM) has been reported by FSRS^32^. Although the backbone of DCM is slightly different from stilbene (pyran ring replaces the phenyl in the acceptor side), no major changes in the skeletal vibrational modes in the frequency range of 1550–1650 cm^−1^ have been interpreted as the twist of the electron-donating dimethylamino group with the ICT dynamics. The twisted ICT dynamics of a stilbazolium dye has also been reported recently by FSRS, where the twist of diethylaminophenyl group in the stilbene-like backbone (acceptor side phenyl is replaced with pyridinium) with the ICT is evidenced by the appearance of the ethylenic ν_C=C_ at 1650 cm^−1^^90^. Although the detailed changes in the vibrational coupling between the ν_8a_ of phenyl and the ν_C=C_ of ethylene were not resolved, the twist in the stilbene-like backbone of the stilbazolium dye was proposed based on the TDDFT simulations.

Notably, the structural changes of nitroaromatic *push–pull* chromophores, DNBP and DNS are distinct from other chromophores of strong ICT character with similar electron donor groups such as dimethylamino or diethylamino group. While the twist of the electron-donating moieties, dimethylamino of DCM or diethylaminophenyl of the stilbazolium dye have been suggested for the structural changes with the ICT in the excited state^32,90^, the skeletal vibrational modes of stilbene backbone provided clear evidence for the rotation of electron-accepting nitrophenyl group with the ICT of DNBP and DNS in the present study with FSRS. The strong electron-withdrawing character of a nitro group compared to dicyanomethylene of DCM and methyl pyridinium of stilbazolium dye may result in the rotation of the nitrophenyl group in the excited state, which leads to substantial Stokes shifts of 7390–11,300 cm^−1^ in polar solvents. Figs. S11 and S12 in the Supplementary Information show the electron density distributions of DNBP and DNS, respectively, in the highest occupied molecular orbital (HOMO) and lowest unoccupied molecular orbital (LUMO) levels. With the twisted nitrophenyl geometry, the electron densities of HOMO and LUMO levels for DNBP and DNS show spatial separation between the electron-donating dimethylaminophenyl and the electron-accepting nitrophenyl moieties, which strongly supports the twist of nitrophenyl group upon the ICT in the S_1_ excited state^91,92^.

## Conclusion

Highly symmetric in-plane ring stretching (ν_8a_) of the phenyl and the stretching (ν_C=C_) of the ethylene are used for the sensitive measure of the internal rotation accompanying the ICT of the *push–pull* nitroaromatic chromophores. When the biphenyl or stilbene backbones show the structural changes from a more or less planar geometry to a perpendicular geometry by the twist of nitrophenyl group, strong vibrational couplings between the ν_8a_ and ν_C=C_ modes in the frequency range of 1550–1650 cm^−1^ disappear, which results in the ultrafast changes of 220–480 fs in these skeletal vibrational modes of ν_8a_ and ν_C=C_. While the TDDFT simulations can only provide rough estimates for the structural changes of chromophores accompanying the ultrafast ICT process, the time-dependent vibrational probe of the FSRS can provide more precise experimental pieces of information which would be helpful for the further development of theoretical methods.

## Methods

### General

DNBP and DNS (TCI Chemicals, Tokyo, Japan), and all the solvents were used without further purification. The steady-state absorption spectra were measured in a commercial UV/Vis absorption spectrometer (Mega-900, Scinco, Seoul, Korea) and the steady-state emission spectra were obtained with a home-built time-correlated single photon counting (TCSPC) setup based on a TCSPC module (Picoharp 300, PicoQuant, Berlin, Germany) with 405 nm excitation (P–C-405, PicoQuant)^46,93^.

### Femtosecond stimulated Raman spectroscopy (FSRS) setup

The details of FSRS setup used for time-resolved Raman measurements based on a 1 kHz Ti:sapphire regenerative amplifier were described elsewhere^62,63^. The actinic pump pulses (403 nm, ~ 60 nJ/pulse) were generated by second harmonic generation (SHG) in β-barium borate (BBO) crystal and compressed by a pair of chirped mirrors (− 25 ± 10 fs^2^ group delay dispersion; Layertec GmbH, Mellingen, Germany). The narrowband picosecond Raman pump (802 nm, ~ 600 nJ/pulse, < 10 cm^−1^) and the broadband Raman probe pulses (840–980 nm) were combined with the actinic pump pulses at the sample position. The Raman probe pulses were focused with a beam diameter of 50 μm at the sample, while the Raman pump and actinic pump pulses were less tightly focused with the beam diameters of 80–90 μm. The modulations in the Raman probe pulses were detected in a fast charge coupled device detector attached to an *f* = 320 mm spectrograph. The samples were recirculated in a 0.5 mm thick quartz flow cell by a peristaltic pump to minimize photodamage.

### Transient absorption setup

The details of the transient absorption setup based on a 1 kHz Ti:sapphire regenerative amplifier were available elshwhere^41,94^. The actinic pulses at 403 nm (~ 50 nJ/pulse) were generated by SHG in a 0.1 mm thick BBO crystal (A-star photonics, Fuzhou, China) and compressed by a pair of chirped mirrors (− 30 ± 10 fs^2^ group delay dispersion; Layertec GmbH). The whitelight probe pulses were generated by supercontinuum generation in a sapphire window (3 mm thick, Eksma Optics, Vilnius, Lithuania) and filtered by a shortpass filter to remove the fundamental pulses. The actinic pump and whitelight probe pulses are combined at the sample position and the liquid sample in a 0.5 mm thick quartz cuvette was recirculated by a peristaltic pump. The probe pulses were measured in a compact CCD spectrometer (QE65 Pro, Ocean Optics, Largo, FL, USA) and an optical chopper (MC2000, Thorlabs, Newton, NJ, USA) was used to modulate the pump pulses. The instrument response function (IRF) of the transient absorption measurements were determined as ~ 60 fs in the cross-correlation measurements between the actinic pump and probe pulses.

### Density functional theory (DFT) simulations

Time-dependent DFT simulations for the optimized geometries and vibrational spectra were done by the Gaussian 09 software (Gaussian, Inc., Wallingford, CT, USA)^95^. The B3LYP exchange–correlation functional and 6-311G(d,p) basis set were used with the polarizable continuum model for solvent effects. All the vibrational frequencies from the DFT simulations were rescaled by a factor of 0.967^96,97^.

## Supplementary Information

Supplementary Information.
