# Supplementary material for: Twisted intramolecular charge transfer of nitroaromatic push–pull chromophores
Source: Sci Rep. 2022 Apr 21;12:6557. doi: 10.1038/s41598-022-10565-6 (PMC9023442; doi:10.1038/s41598-022-10565-6)
Supplement: Supplementary file 1 — Supplementary Information. [file 41598_2022_10565_MOESM1_ESM.pdf]

[Supplementary Information]

Twisted Intramolecular Charge Transfer of Nitroaromatic *Push-pull* Chromophores

Sebok Lee, Myungsam Jen, Taehyung Jang, Gisang Lee, and Yoonsoo Pang*

Department of Chemistry, Gwangju Institute of Science and Technology, 123 Cheomdangwagi-ro, Buk-gu, Gwangju 61005, Republic of Korea

Table of Contents

1. Steady-state absorption and emission spectra of DNBP and DNS 2

2. Femtosecond stimulated Raman results of DNBP and DNS in $CHCl_3$ 3

3. DFT and TDDFT simulations for DNBP and DNS in $CHCl_3$ 5

4. Exponential fit of time-resolved Raman data with coherent oscillations 20

5. Kinetic analysis between the $\nu_{8a,sym}$ and $\nu_{8a,asym}$ modes of DNBP and DNS 24

REFERENCES 25

*Authors to whom correspondence should be addressed. E-mail address: ypang@gist.ac.kr.

1. Steady-state absorption and emission spectra of DNBP and DNS

The emission spectra of DNBP and DNS in DMSO and acetonitrile solutions were obtained with a near-infrared PMT (R5509-72, Hamamatsu Photonics, Japan) to measure near-infrared emission bands precisely.

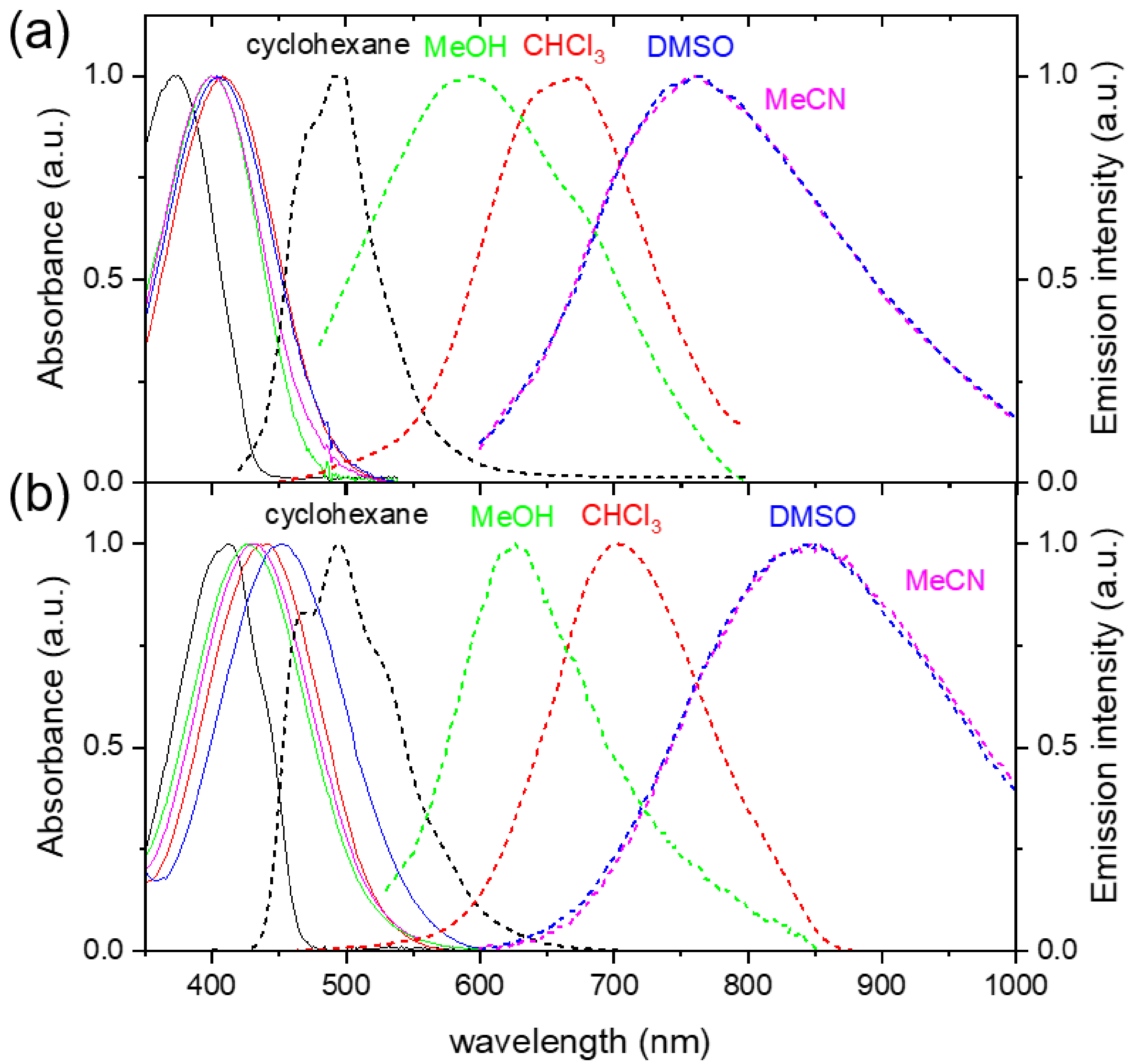

Figure S1. Steady-state absorption (solid) and emission (dotted) spectra of DNBP and DNS in several solvents of varying polarity. The 405 nm excitation was used for the emission measurements.

2. Femtosecond stimulated Raman results of DNBP and DNS in $CHCl_3$

The femtosecond stimulated Raman spectra of DNBP and DNS in $CHCl_3$ solution obtained with the 403 nm excitation are shown in Figure S2. Although the most of the transient absorption background signals included in the Raman probe pulses are subtracted by obtaining the difference spectrum between the excited spectrum at the specific time delay and the ground spectrum taken at the delay of -5 ps of DNBP and DNS, the long-wavelength tails of the stimulated emission still remain mainly in the frequency range of 900-1700 cm^{-1}. The low-order polynomial functions were used to fit the fluorescence backgrounds of DNBP and DNS, as shown in dotted lines in Figure S2(a) and S2(b), respectively. Upon photoexcitation, the major vibrational modes in the frequency region of 1300-1650 cm^{-1} showed the ultrafast spectral changes upon the ICT and subsequent vibrational relaxations.

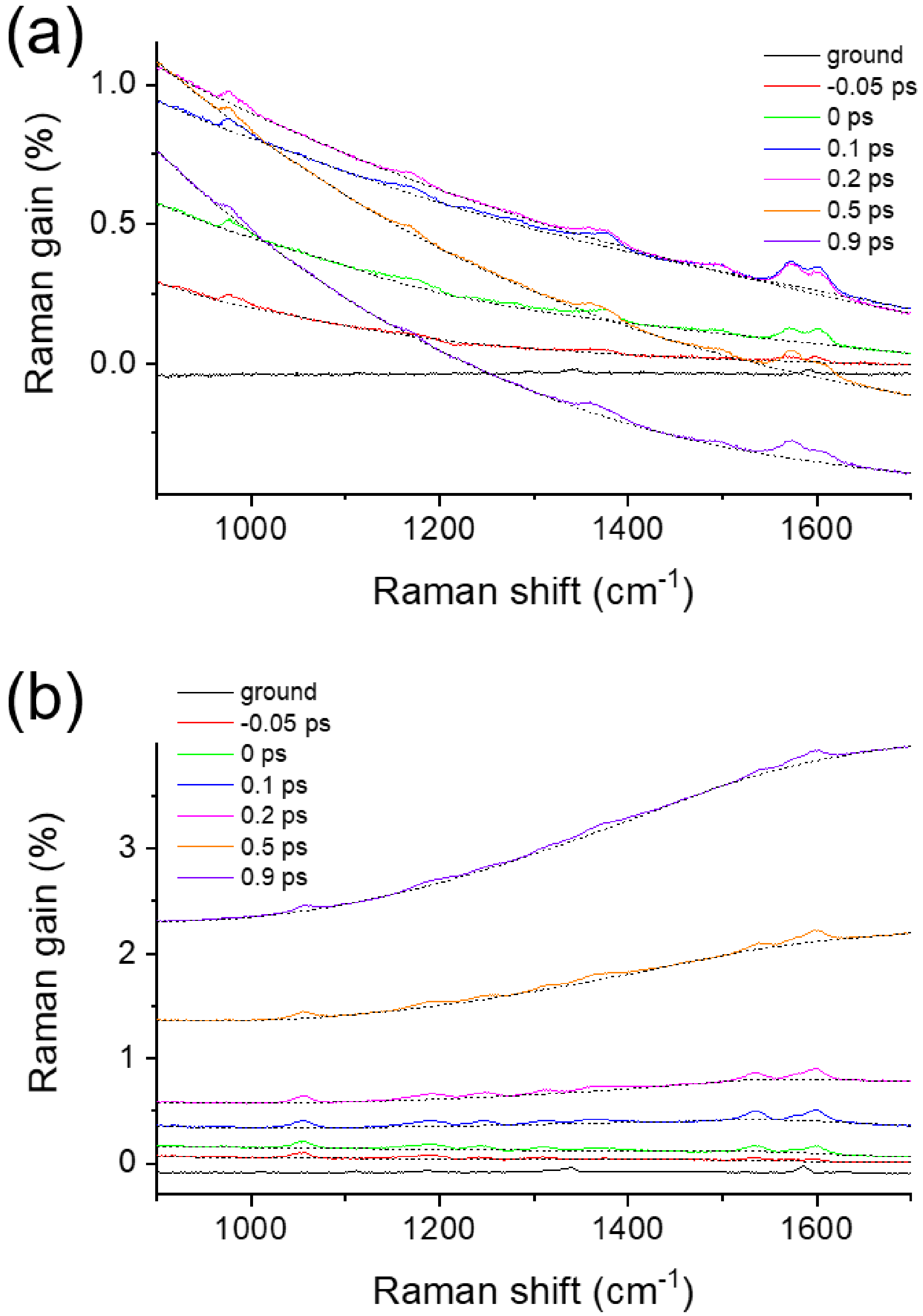

Figure S2. The transient absorption backgrounds in the femtosecond stimulated Raman spectra of (a) DNBP and (b) DNS with 403 nm excitation were removed by the low-order polynomial fit functions.

3. DFT and TDDFT simulations for DNBP and DNS in $CHCl_3$

The optimized structures of DNBP and DNS in $CHCl_3$ in the ground state were obtained by the DFT simulations at B3LYP/6-311G(d,p) level with the polarized continuum model (PCM). Figure S3 represents the optimized structures of DNBP and DNS in the ground state. The dihedral angle between the two phenyl groups of DNBP is estimated as 30.4° and the dihedral angles between the dimethylamino and ethylene, and the nitrophenyl and ethylene are obtained as 0°. The

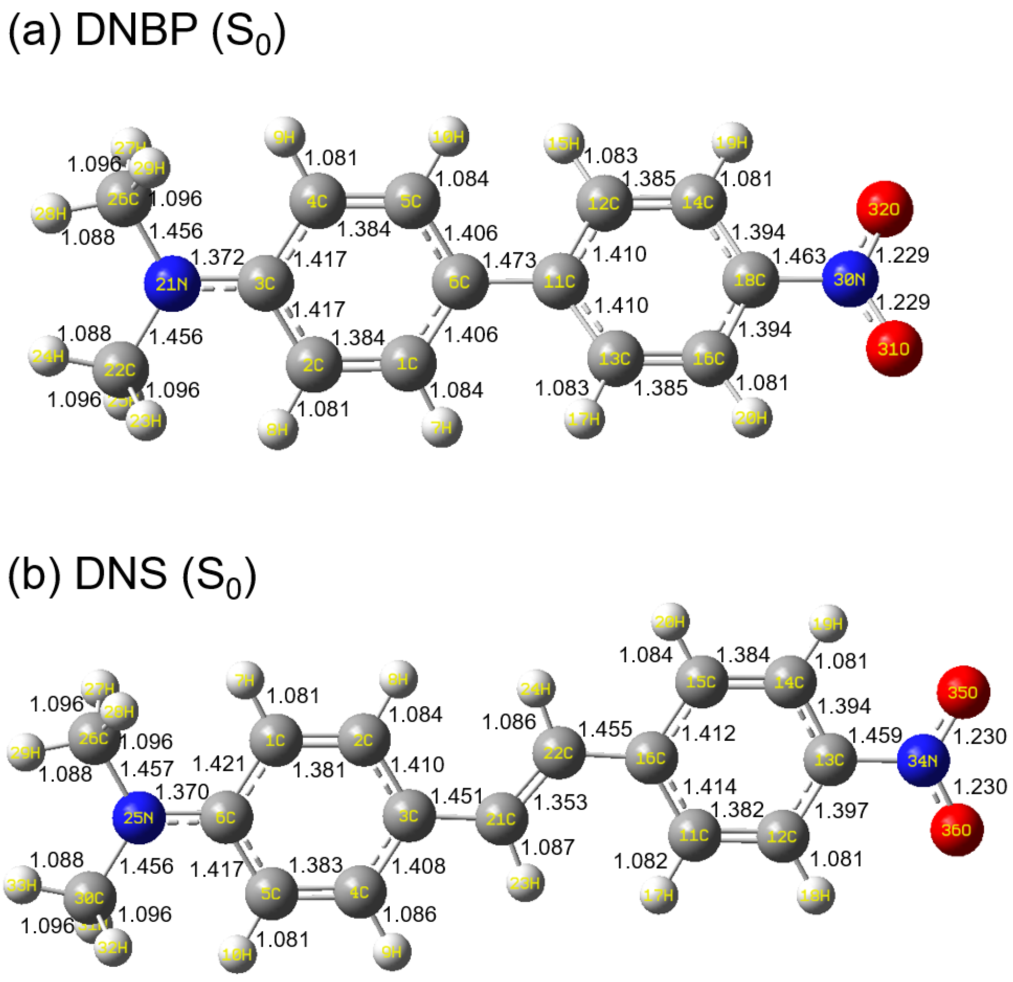

Figure S3. The optimized structures of (a) DNBP and (b) DNS in the ground state (S_0) obtained from the DFT simulations at the B3LYP/6-311G(d,p) level.

structural information of DNBP and DNS including the major bond angles and dihedral angles are

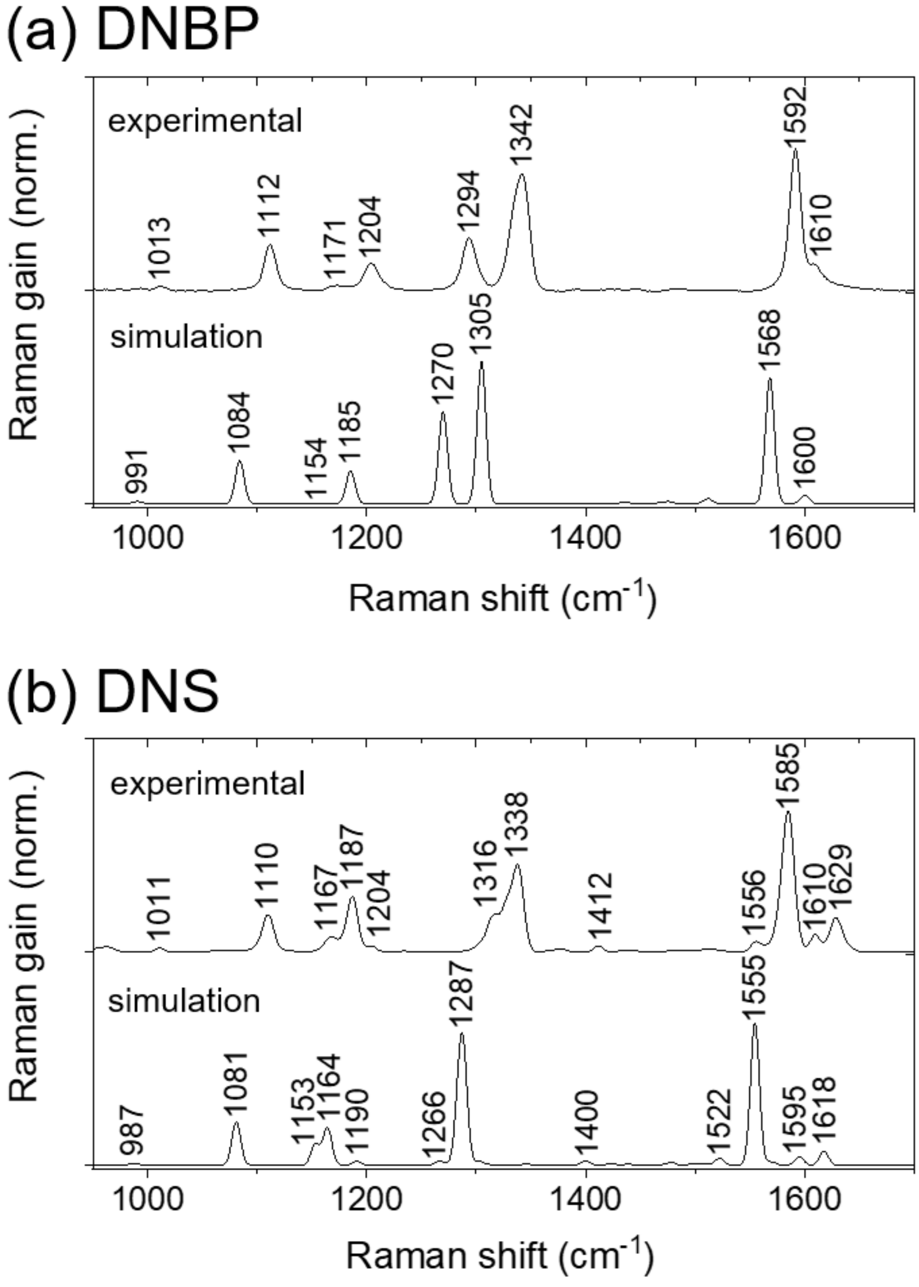

Figure S4. Ground state Raman spectra (experimental vs. simulation) of (a) DNBP and (b) DNS in $CHCl_3$ solution; the The DFT simulations at B3LYP/6-311G(d,p) level with PCM-$CHCl_3$ were used and the vibrational assignments of DNBP and DNS are shown in Figure S6 and S7, respectively, and summarized in Table S1.

summarized in comparison to the excited state values in Table S2.

The ground Raman spectra of DNBP and DNS in $CHCl_3$ were obtained by the DFT simulations at the B3LYP/6-311G(d,p) level with the PCM for $CHCl_3$. The experimental Raman spectra of DNBP and DNS in ground state and the simulation spectra by the DFT simulations are compared in in Figure S4. The vibrational mode assignments of DNBP and DNS in the ground state were based on the DFT simulation results, and summarized as the major vibrational modes shown in Figures S5 and S6, respectively. Table S1 summarizes the experimental and theoretical vibrational frequencies of DNBP and DNS in the ground state with the vibrational assignments.

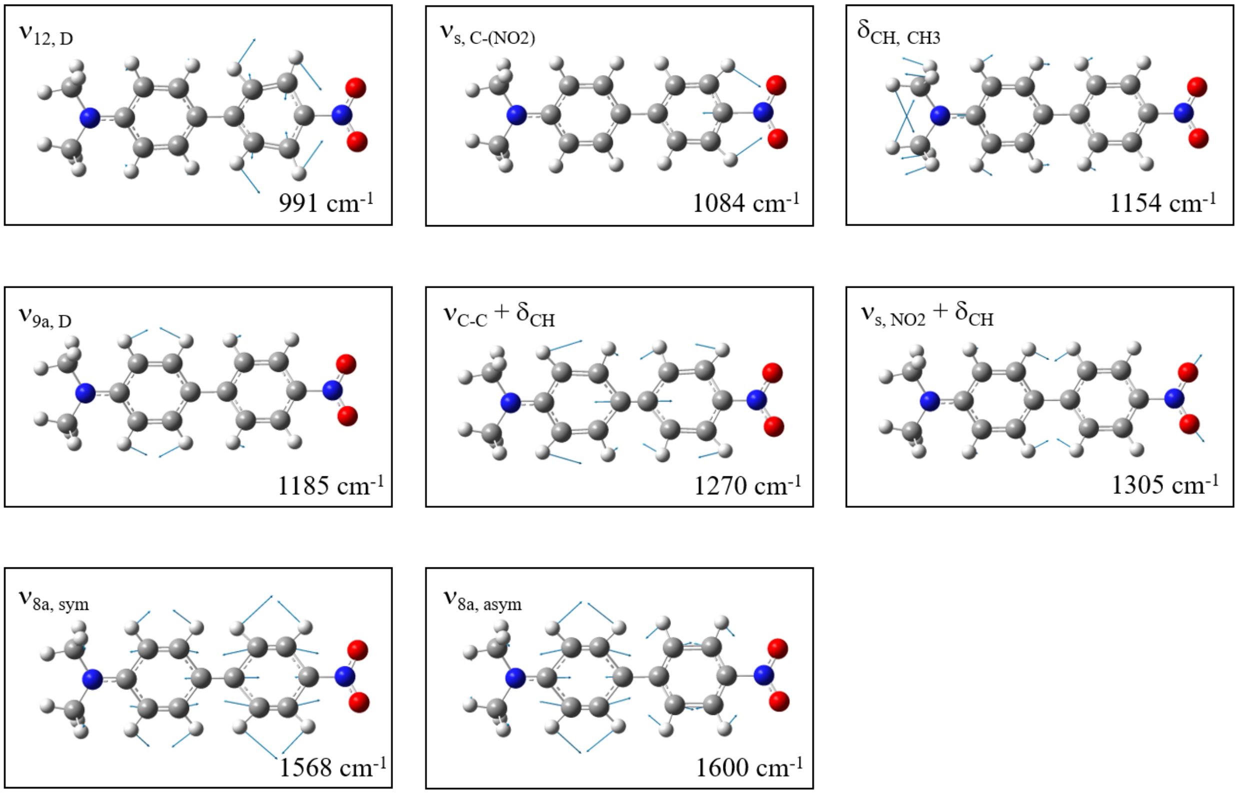

Figure S5. Vibrational normal modes of DNBP in the ground state obtained from the DFT simulation at the B3LYP/6-311G(d,p) level with PCM-$CHCl_3$. The vibrational assignments were summarized in Table S1.

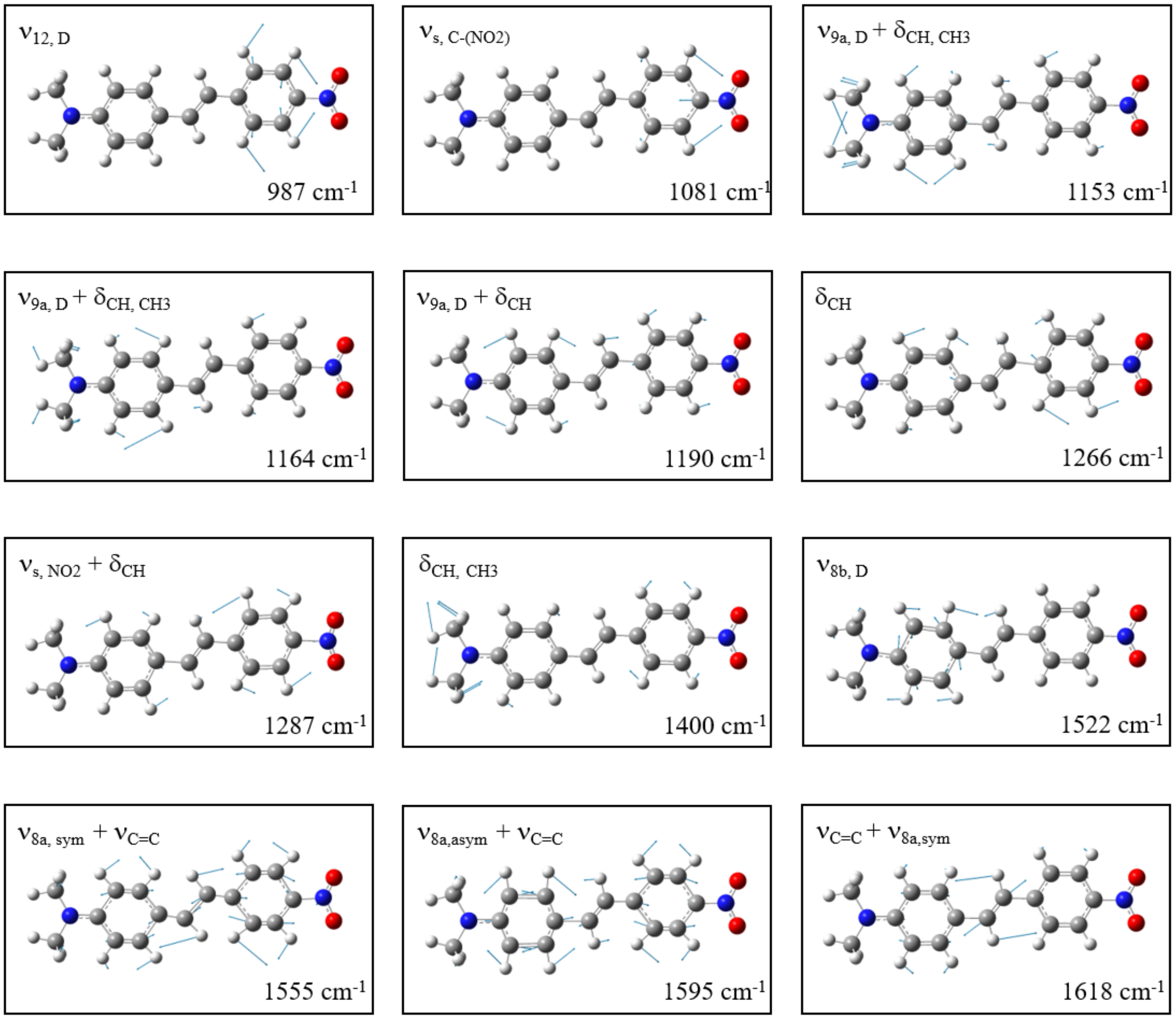

Figure S6. Vibrational normal modes of DNS in the ground state obtained from the DFT simulation at the B3LYP/6-311G(d,p) level with PCM-$CHCl_3$. The vibrational assignments were summarized in Table S1.

Table S1. Vibrational assignments of DNBP and DNS in the ground state

frequency (cm^{-1})		Vibrational assignments[2,3]
experimental	calculated[1]	
DNBP in $CHCl_3$		
1013	991	$\nu_{12, D}$
1112	1084	$\nu_{s, C-(NO2)}$
1171	1154	$\delta_{CH, CH3}$
1204	1185	$\nu_{9a, D}$
1294	1270	$\nu_{C-C} + \delta_{CH}$
1342	1305	$\nu_{s, NO2} + \delta_{CH}$
1592	1568	$\nu_{8a, sym}$
1610	1600	$\nu_{8a, asym}$
DNS in $CHCl_3$		
1011	987	$\nu_{12, D}$
1110	1081	$\nu_{s, C-(NO2)}$
1167	1153	$\nu_{9a, D} + \delta_{CH, CH3}$
1187	1164	$\nu_{9a, D} + \delta_{CH, CH3}$
1204	1190	$\nu_{9a, D} + \delta_{CH}$
1316	1266	δ_{CH}
1338	1287	$\nu_{s, NO2} + \delta_{CH}$
1412	1400	$\delta_{CH, CH3}$
1556	1522	$\nu_{8b, D}$
1585	1555	$\nu_{8a, sym} + \nu_{C=C}$
1610	1595	$\nu_{8a, asym} + \nu_{C=C}$
1629	1618	$\nu_{C=C} + \nu_{8a, sym}$

[1] Calculated vibrational frequencies were rescaled with a factor of 0.967.
[2] The ν and δ denote stretching and bending vibrations, respectively.
[3] The D and A denote the phenyl in the donor (dimethylamino) and acceptor (nitro) sides, respectively.

The pseudo-potential energy surfaces of DNBP and DNS in the ground (S_0) and the first single (S_1) state were obtained by the DFT and TDDFT simulations.[1-3] The ground state geometries of DNBP and DNS was optimized by the DFT simulations at the B3LYP/6-311G(d,p) level with the PCM for $CHCl_3$ with a fixed specific dihedral angles of nitro or nitrophenyl group at a certain value between 0 and 180°. Then, the vertical transition energies were calculated by the TDDFT simulations at the B3LYP/6-311G(d,p) level with the PCM for $CHCl_3$ at each optimized ground state geometry with a specific dihedral angle. The resulting pseudo-potential energy surfaces for DNBP and DNS with the nitro and nitrophenyl rotations are shown in Figure S7. The energy minima for DNBP in the ground state were found with a dihedral angle (30.4°) between two phenyls for the rotation of the nitrophenyl, and with a dihedral angle (0°) between the nitro and phenyl for the rotation of the nitro group. The S_1 minimum of DNBP for the rotation of nitrophenyl group was found with a twisted geometry (dihedral angle between the biphenyl of ~90°) while another for the rotation of nitro group with a planar geometry (dihedral angle between the nitro and phenyl of ~0°). The energy barrier for the rotation of nitro group appears quite high (0.41 eV) and the twist of the nitrophenyl lowers the energy of DNBP in the excited state by 0.18 eV. Further optimization of DNBP in the S_1 excited state with the planar, twisted dimethylamino, twisted nitro, and twisted nitrophenyl geometries were performed by the TDDFT simulations at the B3LYP/6-311G(d,p) level with the PCM for $CHCl_3$ by using the pseudo-geometries obtained with the fixed specific dihedral angles of 0° or 90° in the ground state as the initial geometry. The S_1 minimum was found with the twisted nitrophenyl geometry, where the dihedral angle for the nitrophenyl rotation was found as 89.5° with the relative energy of 2.13 eV from the ground state minimum.

The S_1 minimum with the twisted nitrophenyl appears to be 0.22 eV lower in energy than the more

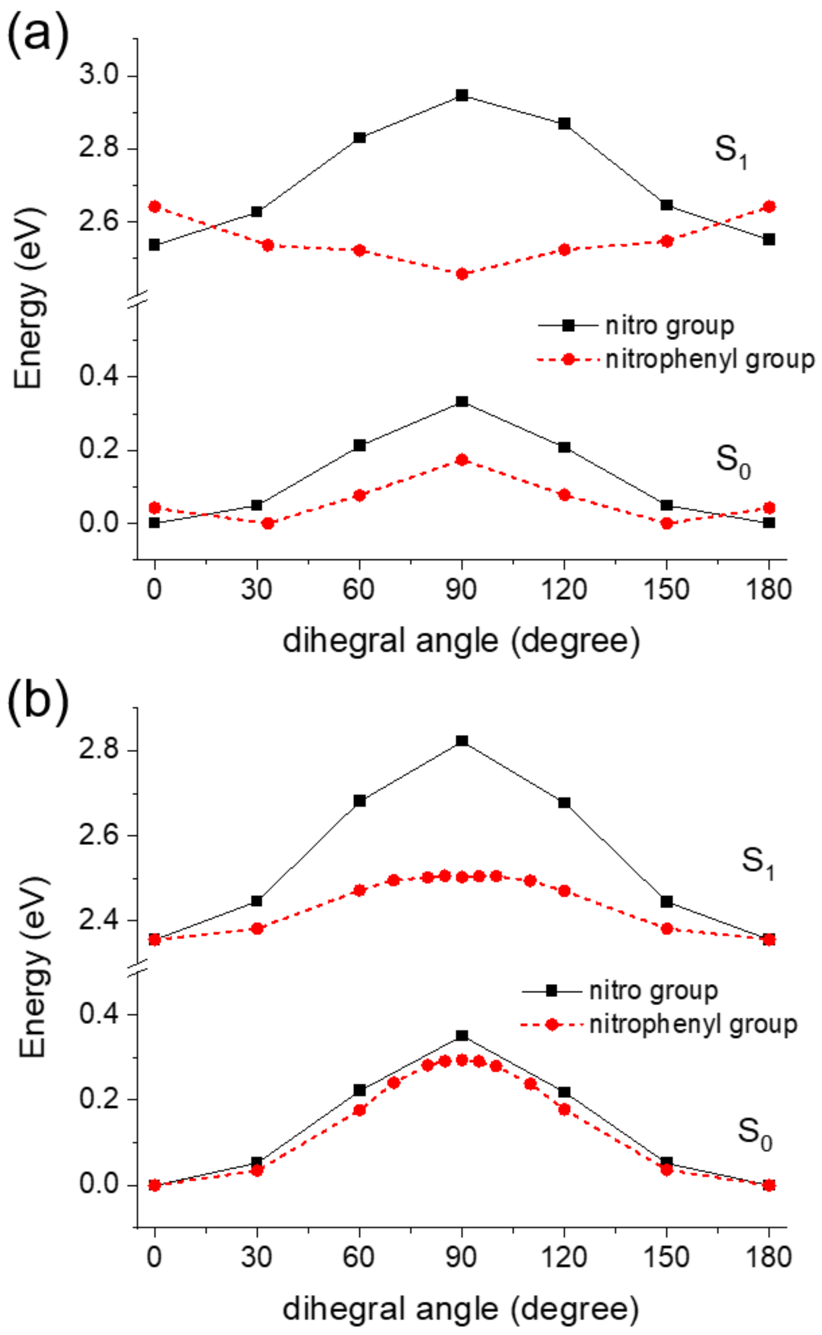

Figure S7. (a) Potential energy curve of (a) DNBP and (b) DNS in the $CHCl_3$ solution based on the ground state optimized geometry and the single point TDDFT simulations at the TD-B3LYP/6-311G(d,p) level; the dihedral rotations along the nitro group and nitrophenyl group were drawn separately.

or less planar geometry in the S_1 state. The isomers of the twisted dimethylamino (2.48 eV) and twisted nitro (2.30 eV) geometry show no major energy stabilization in the TDDFT optimizations. Thus, the Raman spectra of DNBP with the optimized structures in the S_1 excited state with the planar and twisted nitrophenyl geometry were obtained. The optimized structures of DNBP in the S_1 excited state are shown in Figures S8 and summarized in Table S2. The major vibrational modes of DNBP were shown in Figure S10 while Figure 4(b) in the manuscript shows the simulated Raman spectra of DNBP in the S_1 excited state.

For DNS, the ground state minima for the rotation of both nitro and nitrophenyl groups were found with the planar geometry of 0° dihedral angles (for the rotation of the nitrophenyl group between nitrophenyl and ethylene moiety, and for the rotation of the nitro group between the nitro and phenyl). The energy minima of DNS in the S_1 excited state for the rotation of nitro and nitrophenyl groups were also found with the planar geometry of 0° dihedral angles for both nitro and nitrophenyl rotations. The energy barrier of the nitro group rotation appears much higher (0.47 eV) than that of the nitrophenyl rotation (0.15 eV). The previous reports on the excited state molecular geometries of DNS by the TDDFT simulations show that the excited state dynamics of DNS including the twist of nitro or nitrophenyl rotation, and the *trans-cis* isomerization of stilbene backbone, are strongly dependent on solvent polarity.[2-6] In polar solvents, energy barrier for the *trans-cis* isomerization is quite high since the singlet states are stabilized by strong solvent interactions.[3,6] Although several twisted molecular geometries of DNS has been suggested in nonpolar and polar solvents by the theoretical works, the simulation results by the DFT methods, especially for molecules with strong charge transfer characters, can be inaccurate due to the limitation of the DFT methods.[3,7]

Although the relative energy for the isomer with the twisted nitrophenyl group in the S_1 excited state appears higher (0.15 eV) than the S_1 minimum with the planar geometry, a plateau in the dihedral angle of 80-100° for the twist of nitrophenyl group in the pseudo-potential surface in Figure S7(b) may suggest a local minimum with the twisted nitrophenyl group for DNS in the S_1 excited state. The existence of the local minima with the twisted nitrophenyl group have been reported by previous TDDFT simulations in several different environments of vacuum and carbon tetrachloride and dimethyl sulfoxide solution.[2,3] Thus, it would be meaningful to look for the optimized structures of DNS in the local minimum of the twisted dimethylamino, nitro, and nitrophenyl groups to justify the experimental results in the time-resolved Raman measurements of DNS in the excited state.

Similarly, the optimized molecular geometries of DNS in the S_1 excited state for the planar, and twisted dimethylamino, nitro, and nitrophenyl groups were obtained by using the pseudo-geometries obtained with the fixed specific dihedral angles of 0° or 90° in the ground state as the initial geometry. The optimized structure of DNS with the twisted nitrophenyl group (dihedral angle between nitrophenyl and ethylene group is 90.8°) appears located 0.06 eV lower than that with the planar (0.0°) geometry and the twisting of the nitro (72.4°) group lowers the energy of 0.03 eV from the planar isomer in the S_1 state. On the other hand, the twist of the dimethylamino group (90.0°) raises the energy of the S_1 excited state by 0.28 eV from the isomer with the planar geometry. Thus, the optimized structures of DNS in the S_1 excited state with the planar, twisted nitro, and twisted nitrophenyl geometry were used for the Raman spectrum simulations. The optimized structures of DNS in the S_1 excited state by the TDDFT simulations are visualized in Figures S9 and summarized in Table S2. The major vibrational modes of DNS were shown in Figure S10 while Figure 6(b) in the manuscript shows the simulated Raman spectra of DNS in the

S_1 excited state. Further optimization of DNS with the twisted dimethylaminophenyl by the TDDFT method fails to converge in a given time window. Thus, the possibility of the dimethylaminophenyl rotation in the S_1 excited state was not considered for the spectral changes of DNS in the FSRS measurements.

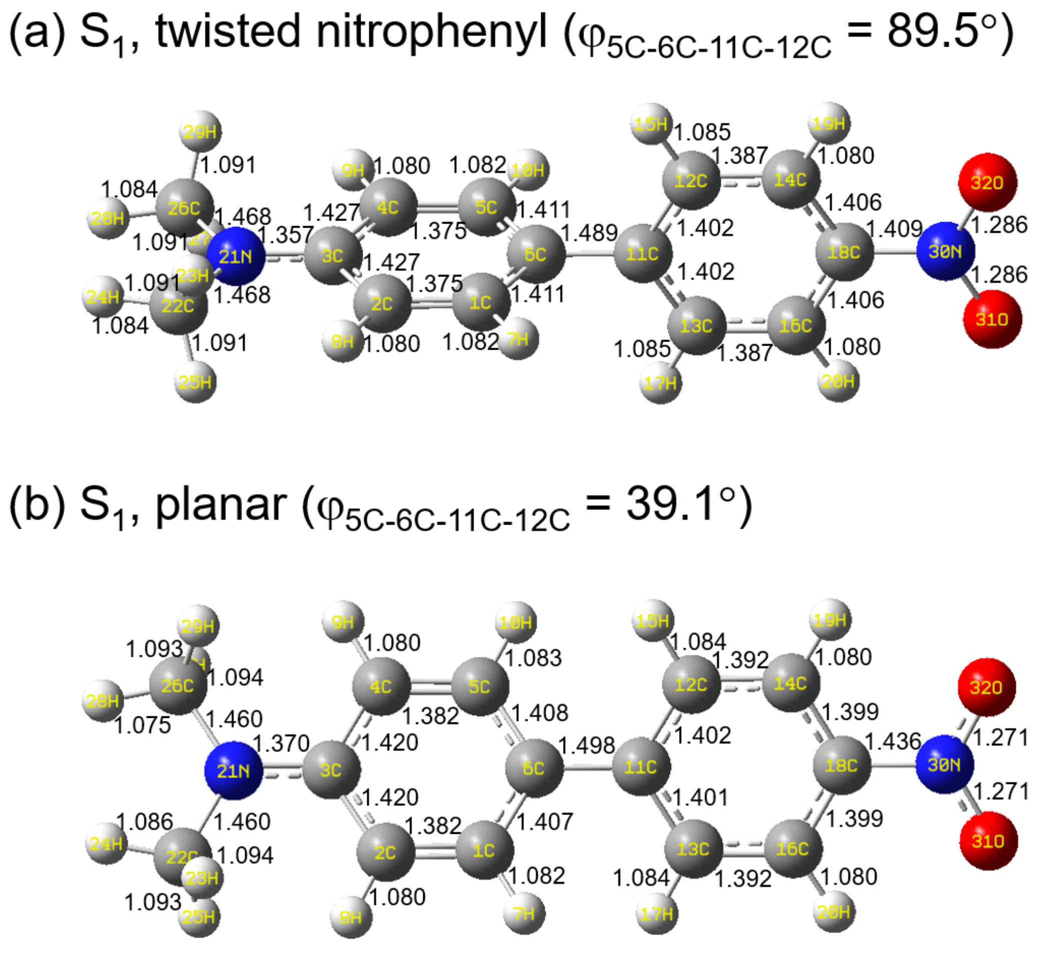

Figure S8. The optimized structures of DNBP in the S_1 excited state with the (a) twisted nitrophenyl and (b) planar geometry obtained from the TDDFT simulations at the B3LYP/6-311G(d,p) level.

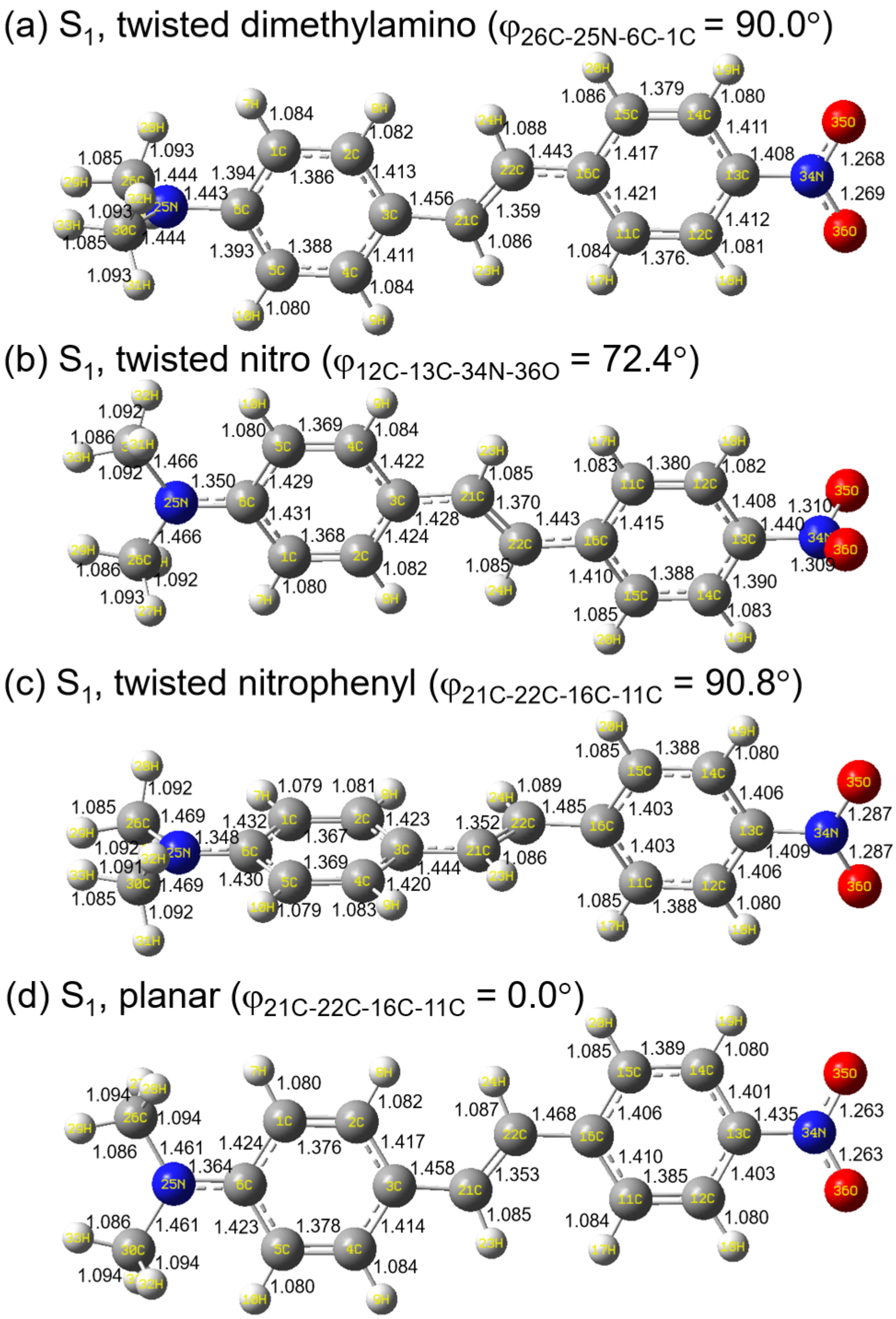

Figure S9. The optimized structures of DNS in the S_1 excited state with the twisted geometry of (a) dimethylamino, (b) nitro, and (c) nitrophenyl group, and (d) the planar geometry from the TDDFT simulations at the B3LYP/6-311G(d,p) level.

Table S2. The optimized structures of DNBP and DNS in the ground state and S_1 excited state with planar and the twisted dimethylamino (DMA), nitro, and nitrophenyl groups

degree of freedom	B3LYP/6-311G(d,p) with PCM-$CHCl_3$				
	ground (S_0)	planar (S_1)	twisted DMA (S_1)	twisted nitro (S_1)	twisted nitrophenyl (S_1)
**1. DNBP**					
**relative energy (eV)**	0.00	2.35	2.48	2.30	2.13
**bond angle (°)**					
32O-30N-31O	123.7	123.4	122.2	123.3	122.5
32O-30N-18C	118.2	118.3	118.9	112.6	118.8
30N-18C-14C	119.4	119.9	120.4	120.1	120.4
14C-18C-16C	121.2	120.1	119.1	119.8	119.2
**dihedral angle (°)**					
5C-6C-11C-12C	30.4	39.1	33.1	26.4	89.5
1C-6C-11C-13C	30.3	30.4	33.1	26.2	89.5
14C-18C-30N-31O	0.4	1.0	0.4	108.7	0.6
16C-18C-30N-32O	0.4	0.7	0.4	74.1	0.6
22C-21N-3C-2C	0.8	0.1	91.8	0.9	0.5
26C-21N-3C-4C	0.8	0.4	91.8	0.9	0.5
**2. DNS**					
**relative energy (eV)**	0.00	2.19	2.47	2.16	2.13
**bond angle (°)**					
35O-34N-36O	123.6	123.1	122.0	123.3	122.4
35O-34N-13C	118.2	118.4	119.1	112.5	118.8
34N-13C-14C	119.5	120.0	120.5	120.1	120.4
14C-13C-12C	121.1	120.3	119.3	120.0	119.1
30C-35N-26C	119.3	118.6	118.0	118.3	121.9
**dihedral angle (°)**					
21C-22C-16C-11C	0.0	0.0	0.0	0.9	90.8
21C-22C-16C-15C	0.0	0.0	0.0	0.5	90.6
14C-13C-34N-35O	0.0	0.0	0.1	106.7	0.4
12C-13C-34N-36O	0.0	0.0	0.1	72.4	0.4
30C-25N-6C-5C	0.0	0.0	89.9	0.3	1.7
26C-25N-6C-1C	0.0	0.0	90.1	0.5	2.1

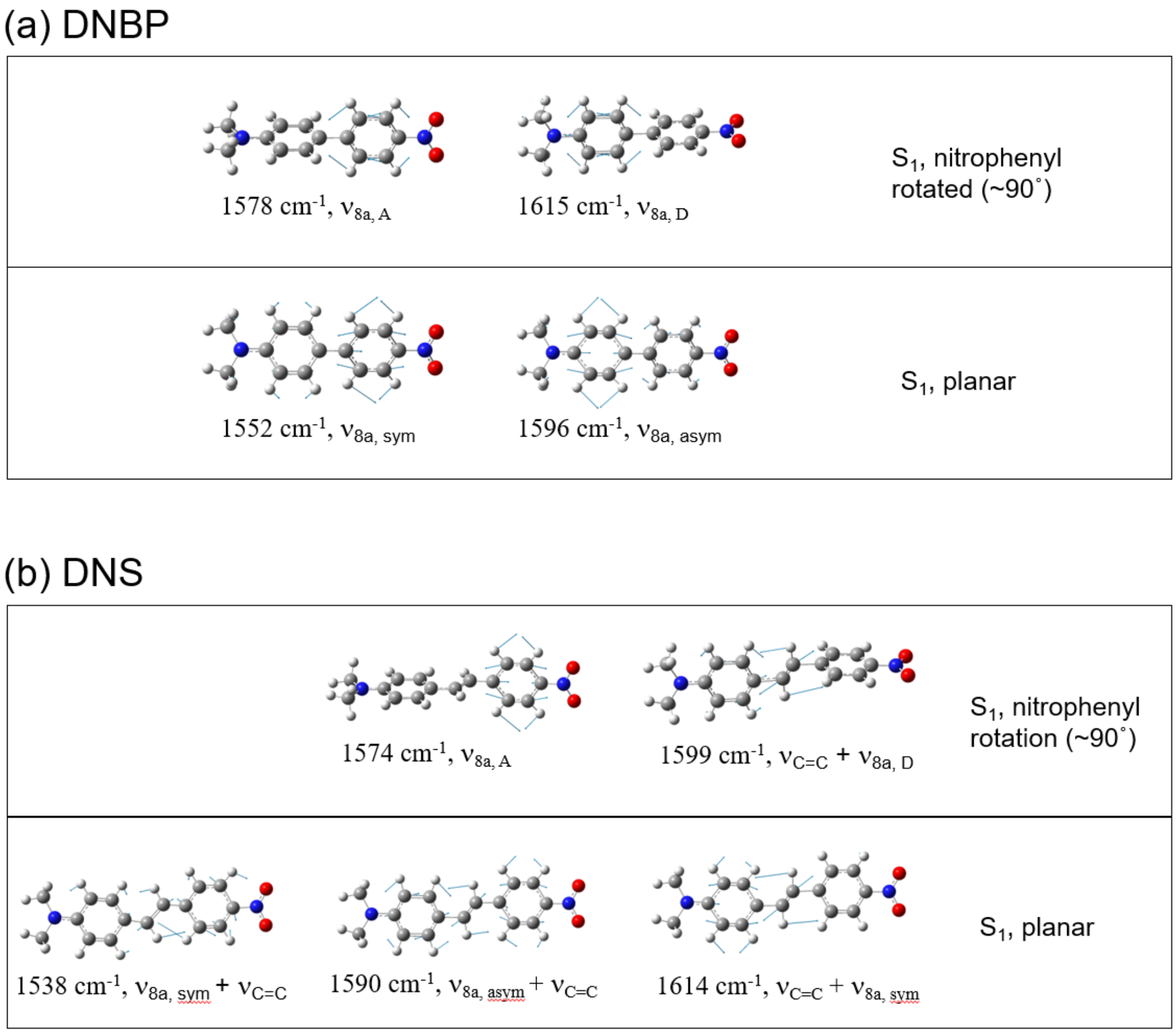

Figure S10. Vibrational normal modes of (a) DNBP and (b) DNS with the twisted nitrophenyl (90.0˚) geometry in the S_1 state obtained from the TDDFT simulations at the B3LYP/6-311G(d,p) level.

Lastly, the frontier molecular orbital diagrams for the highest occupied molecular orbital (HOMO) and lowest unoccupied molecular orbital (LUMO) levels of DNBP and DNS are shown in Figures S11 and S12, respectively.

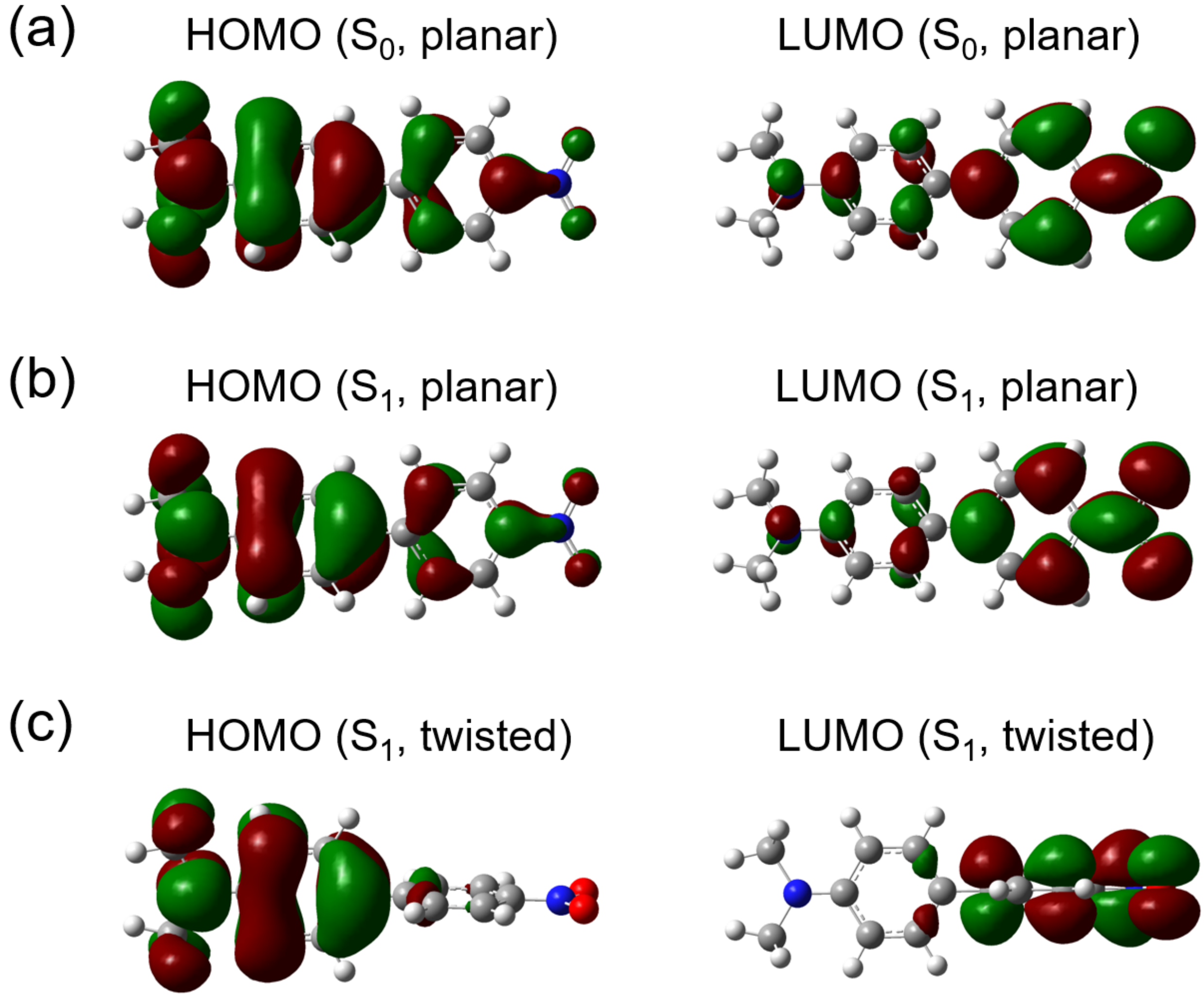

Figure S11. Frontier orbital diagrams for the highest occupied molecular orbital (HOMO) and lowest unoccupied molecular orbitals (LUMO) levels of DNBP with (a) the planar geometry in the ground state, (b) the planar and (c) the nitrophenyl twisted geometry in the S_1 excited state.

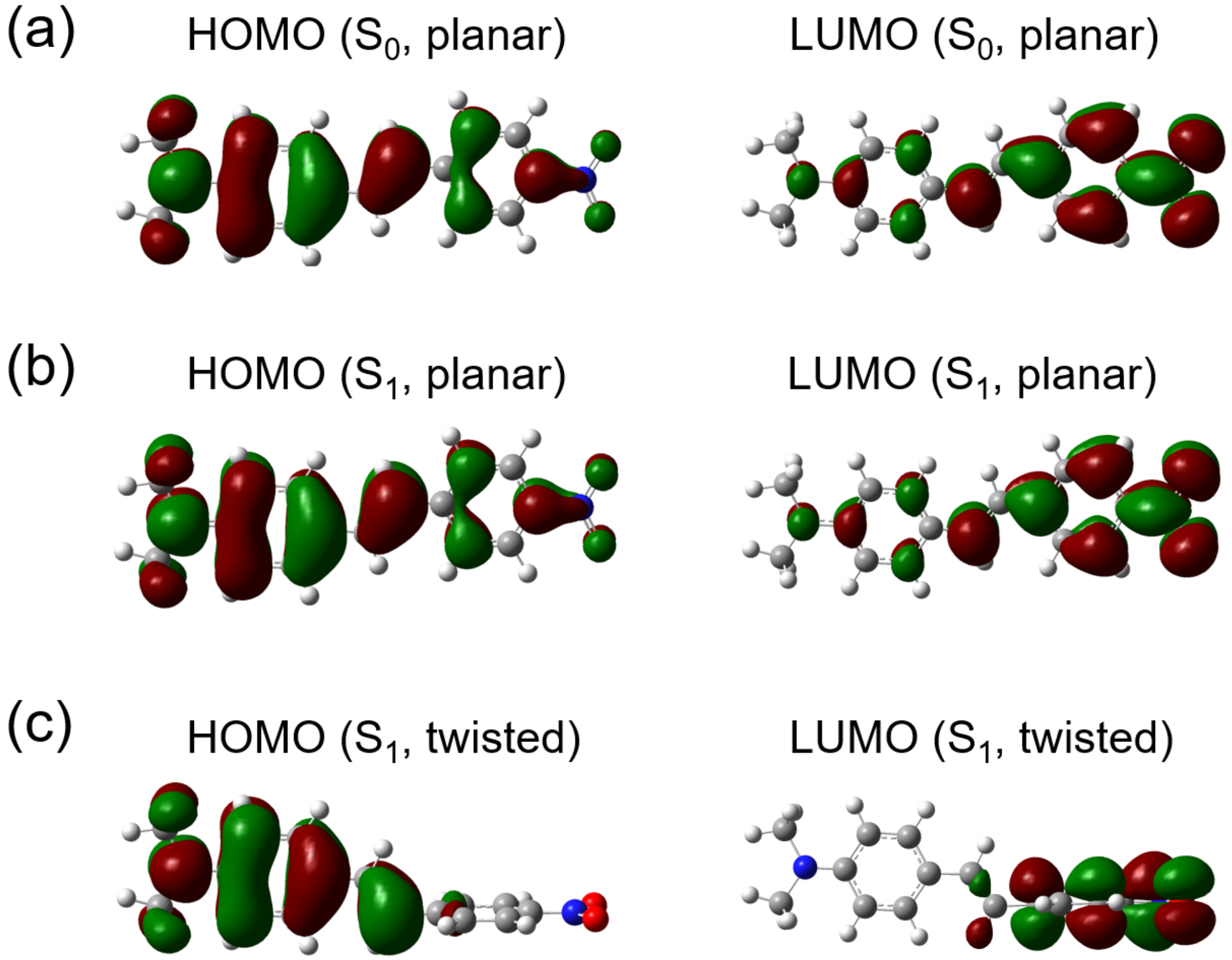

Figure S12. Frontier orbital diagrams for the highest occupied molecular orbital (HOMO) and lowest unoccupied molecular orbitals (LUMO) levels of DNS with (a) the planar geometry in the ground state, (b) the planar and (c) the nitrophenyl twisted geometry in the S_1 excited state.

4. Exponential fit of time-resolved Raman data with coherent oscillations

The excited state vibrational dynamics of DNBP in $CHCl_3$ in the FSRS measurements were fit with the convoluted exponential-Gaussian functions. The Gaussian IRF convoluted with an exponential decay which is also multiplied by a Heaviside step function for the exponential growth and decay model was used,

$$\Delta_{\text{Raman Gain}}(t) = A_0 + \sum_i A_i \exp\left(\frac{\omega^2}{2\tau_i^2} - \frac{t-t_0}{\tau_i}\right)\left[1 - erf\left(\frac{\omega^2 - \tau_i(t-t_0)}{\sqrt{2}\omega\tau_i}\right)\right] \tag{S1}$$

where t_0 is the position of time zero, and ω is the standard deviation of Gaussian function (FWHM = 2.305 · ω) and τ_i is the lifetime of *i*-th kinetic component. Besides, the coherent oscillation signals in the FSRS of DNS in $CHCl_3$ were fit by the sum of the exponential-Gaussian convoluted functions and the several sinusoidal functions multiplied with an exponential damping function,

$$\begin{aligned}\Delta_{\text{Raman Gain}}(t) = A_0 + \sum_i A_i \exp\left(\frac{\omega^2}{2\tau_i^2} - \frac{t-t_0}{\tau_i}\right)\left[1 - erf\left(\frac{\omega^2 - \tau_i(t-t_0)}{\sqrt{2}\omega\tau_i}\right)\right] \\ + \sum_i B_i \exp\left(\frac{\omega^2}{2\tau_{D,i}^2} - \frac{t-t_{osc,0}}{\tau_{D,i}}\right)\left[1 - erf\left(\frac{\omega^2 - \tau_{D,i}(t-t_{osc,0})}{\sqrt{2}\omega\tau_{D,i}}\right)\right]\sin\left(2\pi\frac{t-t_{osc,0}}{\tau_{osc,i}}\right)\end{aligned} \tag{S2}$$

where $t_{osc,0}$ is the position of time zero for the damped oscillation functions, $\tau_{D,i}$ and $\tau_{osc,i}$ are the time constant for damping and the period of each oscillation component, respectively.

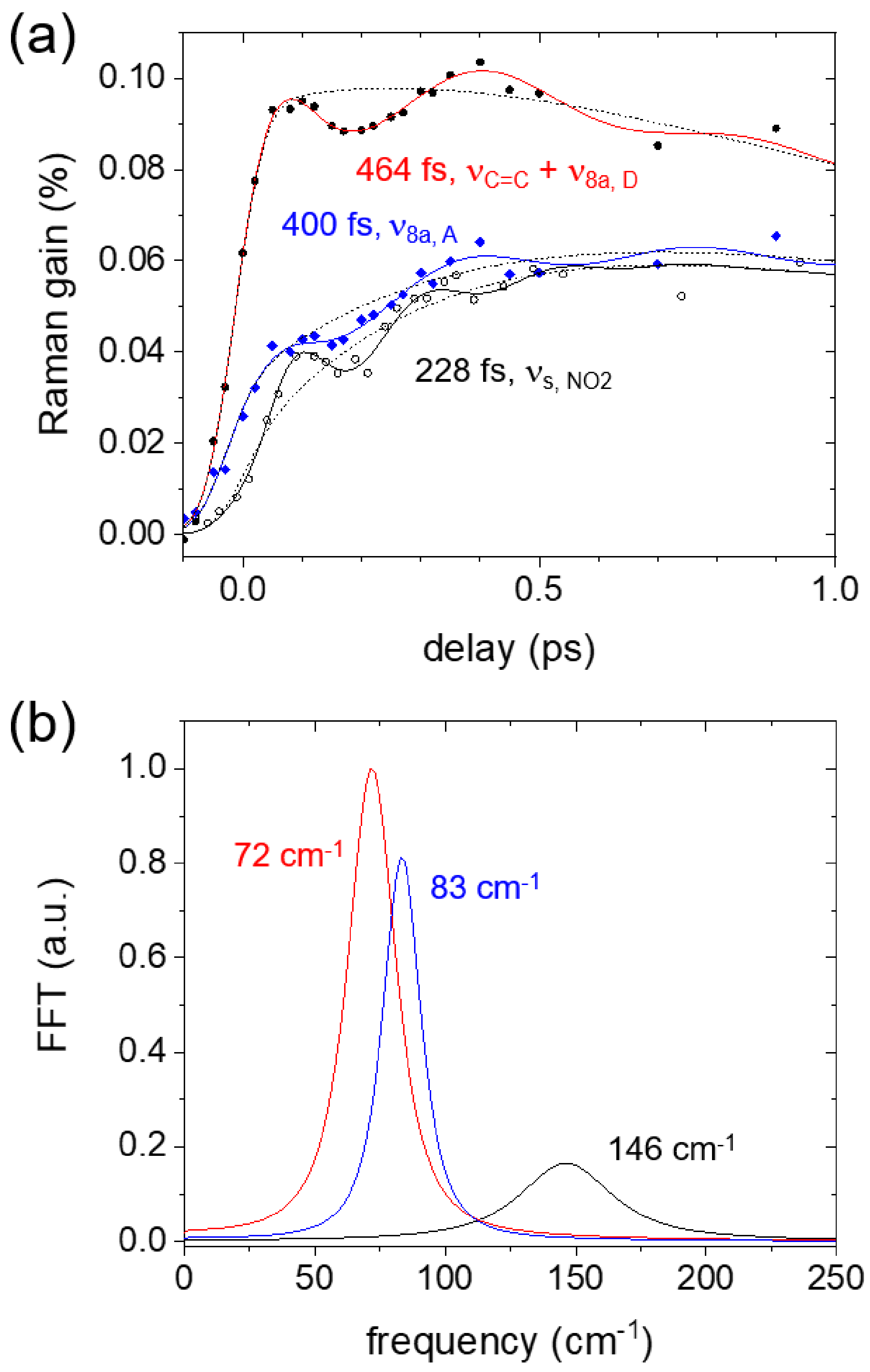

Figure S13. (a) Coherent oscillation signals in the major vibrational modes of DNS in FSRS results, (b) fast Fourier transformation (FFT) results of the coherent oscillation signals.

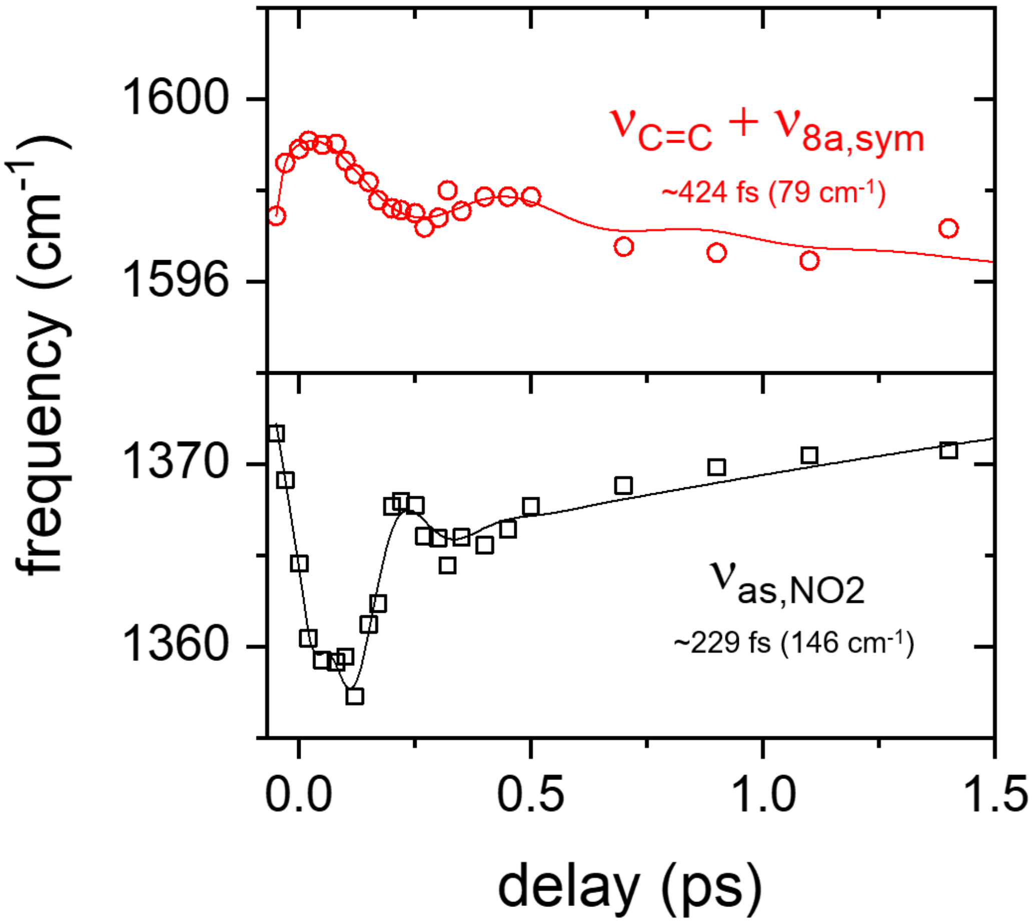

Figure S14. Coherent oscillations in the center frequencies of the $\nu_{C=C} + \nu_{8a,sym}$ and $\nu_{as,NO2}$ modes also shows similar periods of 229 and 424 fs observed from the population dynamics of both modes shown in Figure S12 and Figure 5(c).

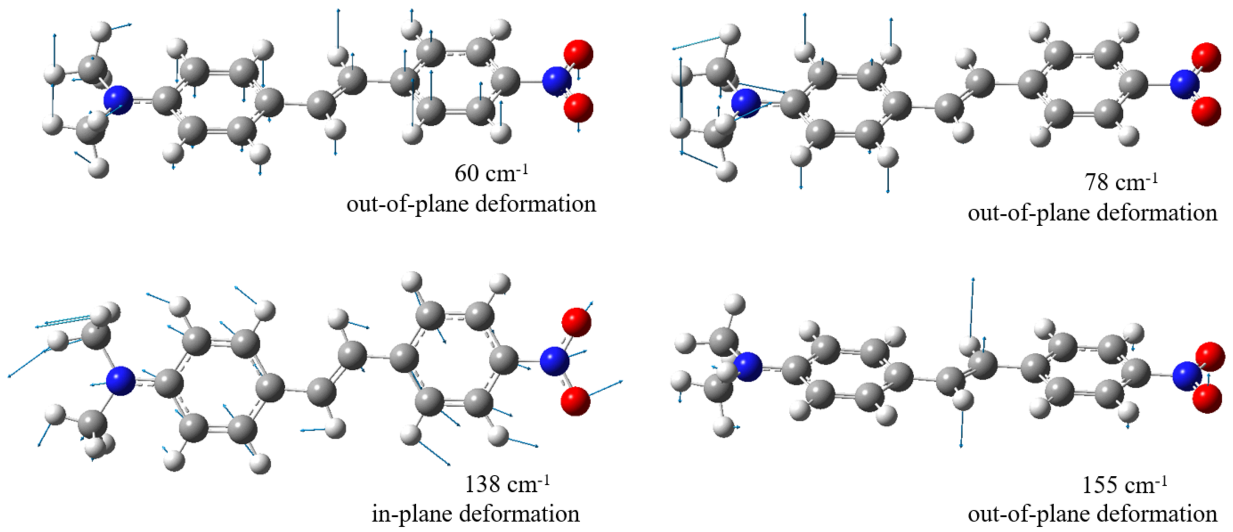

Figure S15. Low frequency out-of-plane deformation and in-plane deformation modes of DNS from the optimized geometry in the S_1 excited state with the planar geometry.

5. Kinetic analysis between the $\nu_{8a,sym}$ and $\nu_{8a,asym}$ modes of DNBP and DNS

The intensity ratios between the $\nu_{8a,asym}$ ($\nu_{8a,D}$) and $\nu_{8a,sym}$ ($\nu_{8a,A}$) modes of DNBP and the $\nu_{C=C}$ + $\nu_{8a,sym}$ ($\nu_{C=C}$ + $\nu_{8a,D}$) and $\nu_{8a,asym}$ + $\nu_{C=C}$ ($\nu_{8a,A}$) modes of DNS were fit with the single exponential decay functions with 220 ± 40 fs (DNBP) and 330 ± 80 fs (DNS) time constants, which represents the ultrafast ICT dynamics in the excited state.

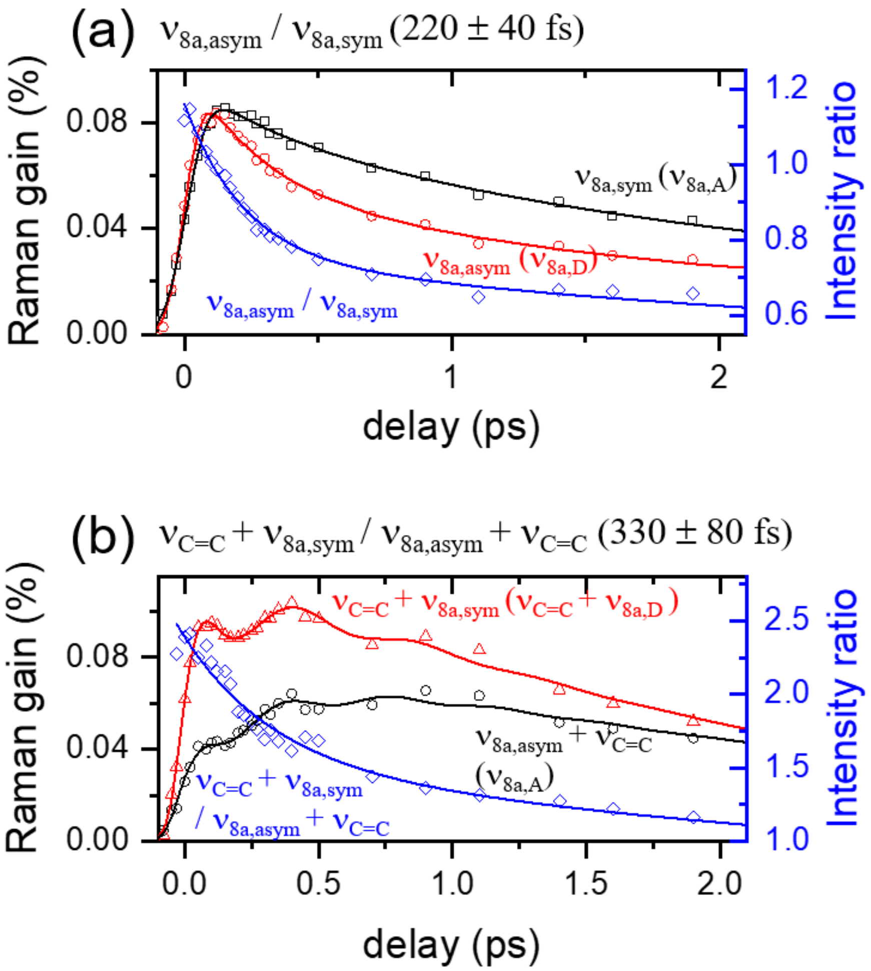

Figure S16. Kinetic analysis for the intensity ratios between (a) the $\nu_{8a,asym}$ ($\nu_{8a,D}$) and $\nu_{8a,sym}$ ($\nu_{8a,A}$) modes of DNBP and (b) the $\nu_{C=C}$ + $\nu_{8a,sym}$ ($\nu_{C=C}$ + $\nu_{8a,D}$) and $\nu_{8a,asym}$ + $\nu_{C=C}$ ($\nu_{8a,A}$) modes of DNS obtained from time-resolved Raman measurements with the 403 nm excitation.

REFERENCES

1 Ghosh, R., Nandi, A. & Palit, D. K. Solvent sensitive intramolecular charge transfer dynamics in the excited states of 4-N,N-dimethylamino-4′-nitrobiphenyl. *Phys. Chem. Chem. Phys.* **18**, 7661-7671, doi:10.1039/C5CP07778H (2016).

2 Singh, C., Ghosh, R., Mondal, J. A. & Palit, D. K. Excited state dynamics of a push-pull stilbene: A femtosecond transient absorption spectroscopic study. *J. Photochem. Photobiol. A* **263**, 50-60 (2013).

3 Rafiq, S. & Sen, P. Dielectric controlled excited state relaxation pathways of a representative push-pull stilbene: A mechanistic study using femtosecond fluorescence up-conversion technique. *J. Chem. Phys.* **138**, 084308, doi:10.1063/1.4792933 (2013).

4 Petsalakis, I. D. *et al.* Theoretical Investigation on the Effect of Protonation on the Absorption and Emission Spectra of Two Amine-Group-Bearing, Red “Push−Pull” Emitters, 4-Dimethylamino-4′-nitrostilbene and 4-(dicyanomethylene)-2-methyl-6-p-(dimethylamino) styryl-4H-pyran, by DFT and TDDFT Calculations. *J. Phys. Chem. A* **114**, 5580-5587, doi:10.1021/jp100338d (2010).

5 Lin, C.-K., Wang, Y.-F., Cheng, Y.-C. & Yang, J.-S. Multisite constrained model of trans-4-(N, N-Dimethylamino)-4′-nitrostilbene for structural elucidation of radiative and nonradiative excited states. *J. Phys. Chem. A* **117**, 3158-3164 (2013).

6 He, Z., Xue, R., Lei, Y., Yu, L. & Zhu, C. Photorelaxation Pathways of 4-(N,N-Dimethylamino)-4'-nitrostilbene Upon S(1) Excitation Revealed by Conical Intersection and Intersystem Crossing Networks. *Molecules* **25**, 2230, doi:10.3390/molecules25092230 (2020).

7 List, N. H., Olsen, J. M., Rocha-Rinza, T., Christiansen, O. & Kongsted, J. Performance of popular XC-functionals for the description of excitation energies in GFP-like chromophore models. *Int. J. Quantum Chem* **112**, 789-800, doi:https://doi.org/10.1002/qua.23059 (2012).
